# A study of the therapeutic mechanism of Jakyakgamcho-Tang about functional dyspepsia through network pharmacology research

**DOI:** 10.7150/ijms.77451

**Published:** 2022-10-17

**Authors:** Woo-gyun Choi, Na Ri Choi, Eun-Jung Park, Byung Joo Kim

**Affiliations:** 1 Division of Longevity and Biofunctional Medicine, School of Korean Medicine, Pusan National University, Yangsan 50612, Republic of Korea.; 2 Department of Food and Nutrition, College of BioNano Technology, Gachon University, Seongnam 13120, Republic of Korea.

**Keywords:** Jakyakgamcho-Tang, Functional dyspepsia, Network pharmacology, Traditional medicine

## Abstract

Herbal medicines have traditionally been used as an effective digestive medicine. However, compared to the effectiveness of Herbal medicines, the treatment mechanism has not been fully identified. To solve this problem, a system-level treatment mechanism of Jakyakgamcho-Tang (JGT), which is used for the treatment of functional dyspepsia (FD), was identified through a network pharmacology study. The two components, paeoniae radix alba and licorice constituting JGT were analyzed based on broad information on chemical and pharmacological properties, confirming 84 active chemical compounds and 84 FD-related targets. The JGT target confirmed the relationship with the regulation of various biological movements as follows: cellular behaviors of muscle and cytokine, calcium ion concentration and homeostasis, calcium- and cytokine-mediated signalings, drug, inflammatory response, neuronal cells, oxidative stress and response to chemical. And the target is enriched in variety FD-related signaling as follows: MAPK, Toll-like receptor, NOD-like receptor, PI3K-Akt, Apoptosis and TNF signaling pathway. These data give a new approach to identifying the molecular mechanisms underlying the digestive effect of JGT.

## Introduction

The characteristic of functional dyspepsia (FD) is clinical syndrome accompanied by long-term or repetitive upper abdominal pain and discomfort even without a fundamental organic disease that can be pointed out as the cause of symptoms 1. Pharmacological treatment for functional dyspepsia is still insufficient 2. Several trials to address this were generally disappointing, with small improvements in *Helicobacter pylori* eradication 3, proton-pump inhibitors 4, and histamine H2-receptor antagonists 5 compared to placebo. Furthermore, despite the poor effects, pharmacological preparations also pose a danger of side effects (e.g. cisapride).

Nevertheless, despite the perception that herbal medicines have few side effects and excellent effects on symptoms, the number of systematic clinical studies available has been limited due to the lack of standardization of herbal medicine ingredients.

Jakyakgamcho-Tang (JGT) is a widely prescribed spasmolytic in traditional chinese medicine, consisting of Radix Paeoniae and *Radix Glycyrrhizae* and, and of indicator substances such as albiflorin, benzoylpaeoniflorin, glycyrrhizin, isoliquiritin, liquiritigenin, and paeoniflorin. And it has the effect of skeletal muscle cramps caused by hemodialysis and nervous stimulation 6,7. It is also reported that JGT is effective in relieving myodynia caused by chemotherapy or peripheral nerve damage, and can alleviate intestinal cramps through smooth muscle relaxation 8,9,10. In particular, *Radix Glycyrrhizae* in JGT contains isoliquiritin and glycyrrhizin, which has a remarkable convulsive effect 11. The muscle-relaxing effect of JGT through the interaction of complex constituents has not yet been clearly identified, but it has fewer side effects than the muscle relaxation effect of the muscle relaxants that directly acts on the central nervous system 11. However, despite many of these clinical effects, understanding of mechanisms is still insufficient, requiring investigation of the pharmacological characteristics of JGT at the systemic level.

Because of the complex pharmacological properties of multi-compound multi-target multi-pathway preparations of traditional herb drugs, investigating a comprehensive mechanism of action with the existing biological experimental methodological approach has fundamental limitations 12-19. With the rapid development of bioinformatics and systems biology, a new research method called network pharmacology has been proposed 20,21. Network pharmacology collects information about biological systems that are discovered through systematic disturbances of biological systems into big data and classifies them into monitoring of gene, protein, and information pathway response. Then apply mathematical models to describe the system structure and its response to individual perturbations 22. Unlike existing pharmacology research strategies based on 'one target, single drug', network pharmacology describes the complexity between among biological systems, drugs and diseases from a network perspective, which shares a holistic philosophy with traditional chinese Medicine 23. Through this, the mechanism of disease and the mechanism of medicine activity are identified through interaction between the constituents of drug and genes, and between genes and disease-related factors 12-19. Until now, these network pharmacology characteristics have wonderfully investigated the poly-pharmacological characteristics of traditional medicines by suggesting that herbal medicines have pharmacological mechanisms and effects (e.g., therapeutic adjustment of biological procedure such as angiogenesis, cell cycle regulation, apoptosis, inflammation, insulin metabolism, reduction, oxidation and proliferation) through interactions between compounds and targets for medical cure of variety diseases, including arthritis, diabetes, ischemic stroke and cancer 12-19,24-34. In addition, the components of JGT were analyzed through a network pharmacology study, and the therapeutic effects for antidepressants, respiratory diseases, and chronic gastritis was confirmed 35-37. Through this network pharmacology study, we try for identify from a system perspective the therapeutic mechanisms underlying the characteristics of digestive properties of JGT.

## Materials and methods

### Sorting of active chemical compounds in Jakyakgamcho-Tang

In order to know the herbal components constituting JGT, information on chemical compounds were searched using the Traditional Chinese Medicine Systems Pharmacology (TCMSP) database 38. And then, TCMSP was used to screen compounds that met previously proposed criteria 70,87,88 based on ADME properties (absorption, distribution, metabolism and excretion) (i.e., oral bioavailability (OB), Caco-2 permeability, and drug-likeness (DL)) 38: OB ≥ 30%, Caco-2 permeability ≥ -0.4, and DL ≥ 0.18. Among these, OB, one of the most important considerations in the design and development of drugs, refers to the ratio of oral administered drug compounds to general circulation 38,39. In general, OB is 30% or more as a standard for effectively absorbing compounds into the living body 38,39. Caco-2 permeability is an essential indicator of intestinal permeability and medicine efflux and is based on the assessment of rate of the absorption and diffusion of compounds through Caco-2 human intestinal cells 38,40-42. The Caco-2 permeability criterion is -0.4, and if it is lower than that, the compound is regarded to be non-permeable in the intestinal epithelium 43,44. DL is a criterion for qualitatively evaluating which specific compounds are structurally and physicochemically suitable for use as a medicine 38,45. As an indicator for active compounds, DL is based on 0.18 or higher, which is usually used as the limitation for determining the pharmacological potential of the compound because the average DL of all medicine is 0.18 38,45.

### Target identification

Target information of the compound was found by searching for TCMSP 38. The target proteins were linked to the official gene name through the UniProtKB database 46.

### Functional enrichment analysis

For perform gene ontology (GO) enrichment analysis, the target gene list was entered into query of g:Profiler and analyzed 47. Pathway enrichment analysis was achieved using Kyoto Encyclopedia of Genes and Genomes (KEGG) database 48. Functional association analysis of the target gene list was performed through GeneMANIA 49.

### Construction of network

First, herbal medicine-active chemical compound (H-C) network was created from the herbal medicines with their active chemical compounds, and the active chemical compound-target (C-T) network was created from the compounds with their targets, finally the target-pathway (T-P) network by connecting the targets with the signaling pathways in which they are enriched. In addition, the Protein-Protein Interaction (PPI) network was created by applying the target gene to the STRING database (interaction confidence score ≥ 0.9) 50. A network consists of nodes and edges that describe the interactions between nodes 51. And the degree is defined as the number of edges on the node 51. The analysis and visualization of the created network was carried out using Cytoscape software 52.

### Contribution index evaluation

In order to evaluate the network- and efficacy-based contribution index (CI) about active chemical compounds of JGT, it was calculated as follows according to the previous procedure 53:



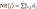





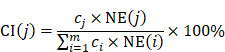



where, *m* represents the number of compounds, *n* represents the number of targets of chemical compound *j*, *d_i_* represents the number of links of target *i* of chemical compound *j*, and *c_i_* (or *c_j_*) represents the number of previous studies in which “functional dyspepsia” and compound *i* (or *j*) are included in the title or abstract from the PubMed database. (https://pubmed.ncbi.nlm.nih.gov/). The chemical compounds with the highest CIs were considered to take on more important role in the pharmacological activity of the herbal medicine 53.

## Results

**Figure 1:**
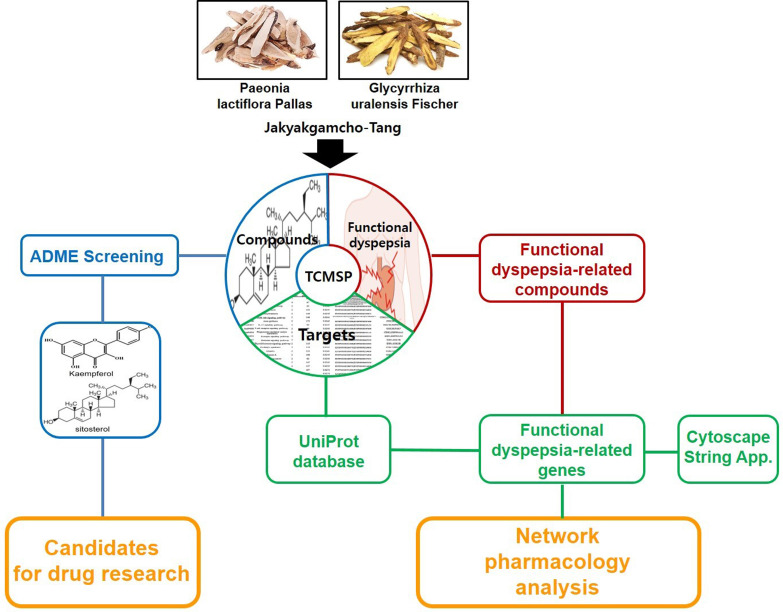
The Schematic diagram of pharmacological network of the digestive mechanisms of Jakyakgamcho-Tang.

The network pharmacology study about mechanism of JGT was carried out as follows (Figure 1). Using TCMSP, the chemical components about the two main herbs of JGT were analyzed to investigate bioactive compounds according to the ADME characteristics and identify targets of active compounds. Then, extensive herbal medicine-related data was integrated into the network and network pharmacology analysis was performed (Figure 1).

### Total chemical compounds and active chemical compounds of Jakyakgamcho-Tang

For comprehensive information of compounds constituting JGT, was acquired through TCMSP 38 (Supplementary Table S1), of which compounds corresponding to with OB ≥ 30%, Caco-2 permeability ≥ -0.4, and DL ≥ 0.18 were defined as active compounds 13,38,53. As a result, it was confirmed that 94 active compounds are present in JGT (Supplementary Table S2).

### Targets of Jakyakgamcho-Tang

We used the TCMSP database to analyze the targets about the active chemical compounds of JGT 38, confirming that there were 73 human FD-associated and 146 non FD-associated targets for JGT (Supplementary Table S3).

### Network pharmacology-based analysis of Jakyakgamcho-Tang

**Figure 2:**
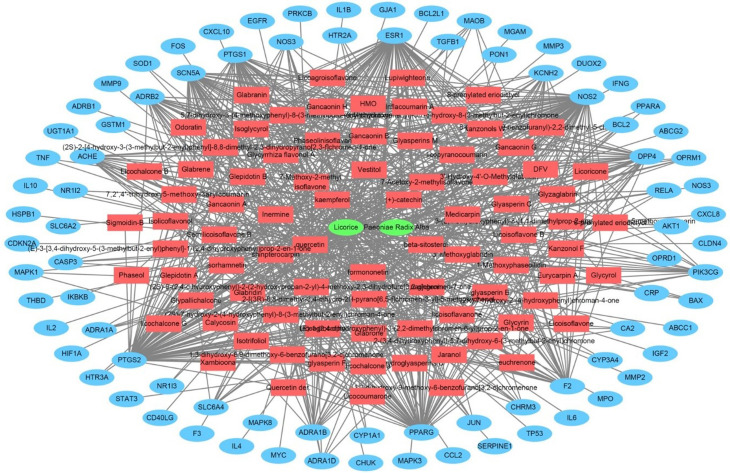
The herbal medicine-active chemical compound-target network of Jakyakgamcho-Tang. Green nodes are herbal medicines; red nodes are active chemical compounds; blue nodes are FD-related targets.

**Figure 3:**
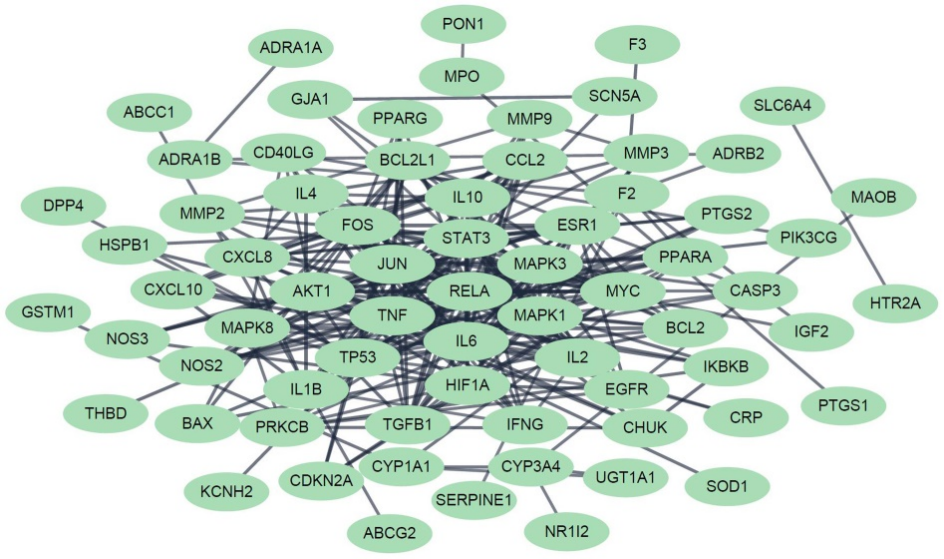
The protein-protein interaction network for the FD-related targets of Jakyakgamcho-Tang. Green nodes are FD-related targets.

In order to perform a network pharmacological analysis about the pharmacological characteristics of JGT, an herbal medicine-active chemical compound-target (H-C-T) network consisting of 170 nodes (herbal medicine = 2, active chemical compound = 84, FD-associated target = 84) and 779 edges (Figure 2 and Supplementary Table S3) was constructed using detailed information on herbs. Among the active compounds, quercetin (target number = 63) and kaempferol (target number = 26) were found as compounds with comparatively many targets (Figure 2 and Supplementary Table S3), indicating that they are active compounds more important for the therapeutic effect of JGT. And it found that there are 42 genes/proteins targeted by two or more active chemical compounds out of all related genes/proteins (Figure 2), indicating that the poly-pharmacological mechanism works. JGT has many targets and the interactions between them play an important role in biological function and therapeutic potential, so to investigate this, we created a PPI network (70 nodes and 265 links) composed of targets of JGT (Figure 3) 54,55. And the degree as a hub was confirmed in the revealed network, we found a specific node with a high degree that has more than twice the average degree as a hub that was found based on the criteria presented in existing network pharmacology studies 56,57. Through the analysis results, it was showed that RELA (degree = 26), JUN (degree = 24), STAT3 (degree = 24), MAPK3 (degree = 22), TNF (degree = 21), MAPK1 (degree = 21), IL6 (degree = 21), TP53 (degree = 19) and AKT1 (degree = 19) act as a hubs (Figure 3), and it was found that they were the main targets related to the digestive activity of JGT. These herbs can be used as potent targets to induce digestive effects by engaging in the regulation of FD-associated processes. NF-kappa-B is a protein formed by binding Rel-like domain-containing proteins NFKB1/p50, NFKB1/p105, NFKB2/p52, REL, RELB and RELA/p65, and is divided into homo- or heterodimeric complex according to binding combinations, of which heterodimeric p65-p50 complex is most abundant. NF-kappa-B exists in almost all cell types and is a pleiotropic transcription factor, initiated by a wide range of stimuli associated to many biological processes such as apoptosis, cell growth, differentiation, immunity, inflammation and tumorigenesis, and corresponds to the endpoint of sign transduction events. This wide range of effects is also expected to affect FD 58. STAT3 acts as a ground-state transcription factor and plays a role about cell proliferation in many types of cells. Cytokines or growth factors such as interleukin (IL)-6 increased by the activation of STAT3 promote stem cell self-regeneration, cell proliferation, and Th17 cell differentiation. As a result of GO analysis **(Biological Process)**, it was confirmed that STAT3 was also related to eating behavior 59,60. MAPK1 and MAPK3 are members of the MAP kinase family. MAP kinases receive variety extracellular signals to regulate variety cell processes such as cell cycle progression, differentiation and proliferation. In particular, MAPK1 and MAPK3 play an essential role in the MAPK signal pathway. By suppressing the MAPK pathway, it can increase intestinal moisture and promote intestinal peristalsis 61. IL-6 is a powerful inducer for acute reactions and is involved in monocyte and lymphocyte differentiation, and in particular, plays an important role about the final differentiation of B-cells into IL-secreting cells 62. Studies on experimental animals have shown that IL-6 plays a partial role in inhibiting food intake and weight due to glucagon-like peptide-1 (GLP-1) receptor stimulation in Central nervous system 63. TNF is an adipokine and a cytokine. TNF is secreted from macrophages and induces cell death of certain cancer cell lines. It is a powerful pyrogen of heat that causes fever via stimulation of interleukin-1 secretion or direct action by itself, and also plays a role in inducing cachexia. Certain conditions may induce cell proliferation or cell differentiation 64. It is also polyhedral as another feature of TNF and plays an essential role in controlling immunity, metabolism, and food intake 65,66,67. AKT1 is one of the three components (AKT1, 2, 3) that make up AKT kinase and is involved in many processes, including cell survival, angiogenesis, growth, metabolism and proliferation 68. AKT controls glucose uptake by intervening the insulin-induced translocation of SLC2A4/GLUT4 glucose transporter in the cell surface 69.

For a more reliable evaluation, we evaluated the pharmacological contribution to the digestive effect of herbal medicines by measuring the CI of active compounds of JGT 53,70. As a result of the evaluation, it was confirmed that naringenin has the highest CI at 98.94%. Naringenin is a compound contained in licorice and is associated with ABCC1, AKT1, BCL2, CASP3, ESR1, MAPK1, MAPK3, PIK3CG, PPARA, PPARG, PTGS1, PTGS2, RELA, SOD1 and UGT1A1 (Figure 2). It was found that the compound contributed significantly to the digestive activity of JGT.

Together these results, we were able to confirm the system-level pharmacological Characteristics of the digestive effect of JGT.

### Functional enrichment investigation about networks of Jakyakgamcho-Tang

**Figure 4:**
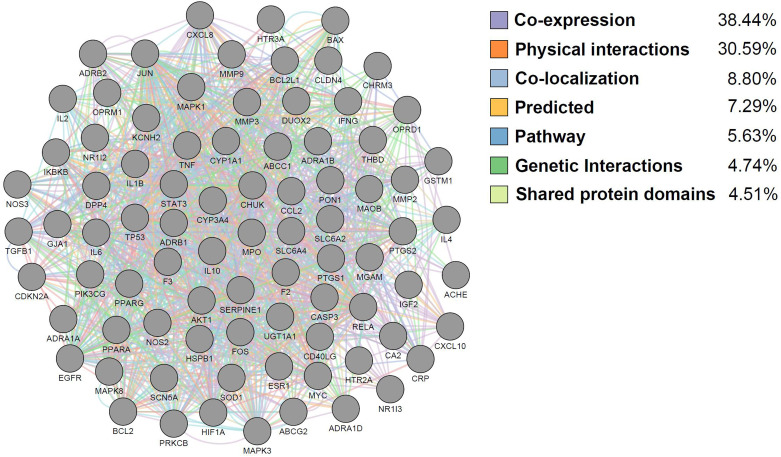
Functional interaction analysis of the FD-related targets of Jakyakgamcho-Tang. Gray nodes are FD-associated targets; colored edges are mechanisms of the function interactions between the targets.

**Figure 5:**
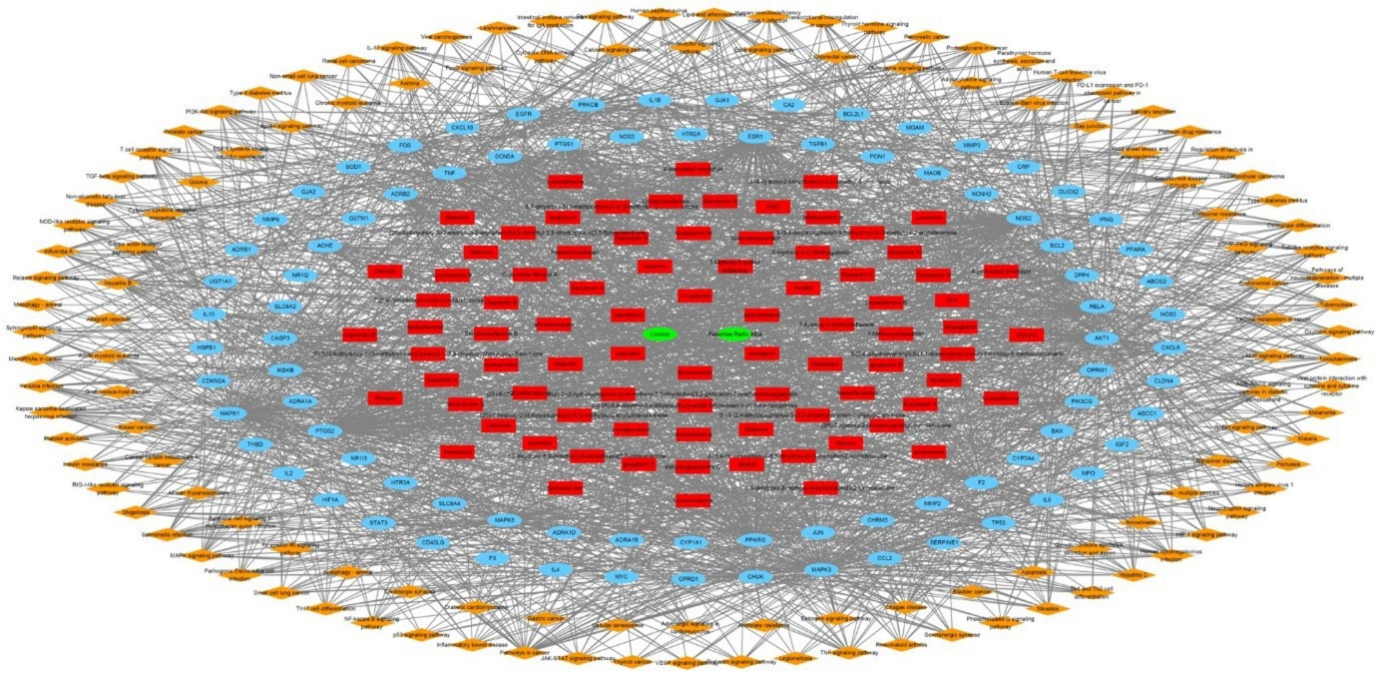
The herbal medicine - active chemical compound - target - pathway network of Jakyakgamcho-Tang. Green nodes are herbal medicines; red nodes are active chemical compounds; blue nodes are FD-related targets; orange nodes are signaling pathways.

The targets of JTG were analyzed using GO enrichment analysis, a method that has been recently spotlighted in investigating molecular mechanisms. The results were concentrated in GO terms related to regulation of various biological activities, including cellular behaviors of muscle and cytokine, calcium ion concentration and homeostasis, calcium- and cytokine-mediated signalings, drug, inflammatory response, neuronal cells, oxidative stress and response to chemical (Supplementary Table S4). And the results are matched with previously reported therapeutic mechanisms of herbal medicines 71,72,8,73,74,75,76,77-80. Furthermore, GeneMANIA analysis showed that JGT targets interact with each other and function through various mechanisms (Figure 4), suggesting the similarity in pharmacological roles. Since it was known that various signaling pathways were related to FD 81-86, we performed pathway enrichment analysis and found following signalings rich in targets of JGT (Figure 5 and Supplementary Table S4). These signalings, which contain well-known digestion-related pathways, can be used as therapeutic targets for alleviating FD. Human T-cell leukemia virus 1 infection, Shigellosis, Yersinia infection, Epstein-Barr virus infection, Salmonella infection, Pathogenic Escherichia coli infection, Human immunodeficiency virus 1 infection, Pancreatic cancer, Colorectal cancer, Hepatocellular carcinoma, Amoebiasis, Inflammatory bowel disease, Gastric cancer, Malaria, Chronic myeloid leukemia, Graft-versus-host disease, Renal cell carcinoma and Type I diabetes mellitus are diseases accompanied by dyspepsia 87-104. Apoptosis, TNF signaling pathway, MAPK signaling pathway, PI3K-Akt signaling pathway, NOD-like receptor signaling pathway, Chemokine signaling pathway, Calcium signaling pathway, NF-kappa B signaling pathway, cAMP signaling pathway, Toll-like receptor signaling pathway, Neurotrophin signaling pathway, Thyroid hormone signaling pathway, Autophagy, Growth hormone synthesis, secretion and action, Gap junction, Salivary secretion and p53 signaling pathway are associated with functions related to digestion, which are involved in the occurrence of FD, and functional regulation can alleviate FD 105-117. Totally, this result confirmed the pathway- and molecular-level mechanisms supporting the therapeutic activity of JGT.

## Discussion

Traditionally used herbal medicine has been used as an effective digestive medicine with excellent therapeutic activity as an alternative to existing medicines under the general recognition that they have fewer side effects. JGT is also a well-known herbal medicine prescribed to relieve stomach cramps and indigestion 8-10. Therefore, network pharmacology research was conducted to identify the therapeutic mechanisms underlying the digestive effect of JGT. First, 84 active chemical compounds of JGT and 84 FD-associated human molecular targets were identified through network pharmacology investigation and ADME evaluation. And enrichment analysis confirmed that the target of JGT was related with the regulation of biological activity, including involving cellular behaviors of muscle and cytokine, calcium ion concentration and homeostasis, calcium- and cytokine-mediated signalings, drug, inflammatory response, neuronal cells, oxidative stress and response to chemical, coincidental with previously reported therapeutic mechanisms of herbal medicines 71,72,8,73,74,75,76,77-80. We further revealed that JGT might target variety digestion signalings to exert its dyspepsia-relieving activity, which involve Apoptosis, MAPK, TNF, PI3K-Akt, Toll-like receptor signaling pathway and NOD-like receptor.

**Figure 6:**
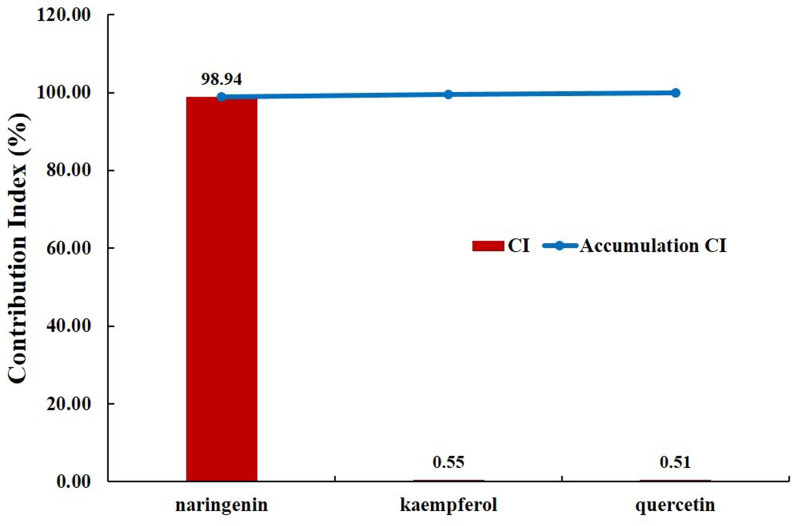
Contribution index analysis for the digestive effect of active chemical compounds of Jakyakgamcho-Tang. A graph depicting the analysis result of contribution index (CI) for the digestive effect of active chemical compounds of JGT. The CI of naringenin was found to be higher than 98%. Note that the CIs of compounds that are not present in the graph is '0'.

There have been reports that chemical components of JGT contribute to digestive activity. quercetin promotes protein absorption through the internalization of digested oligopeptides in the intestinal epithelium Rather than increasing intracellular permeability 118. Kaempferol has been studied to have a positive effect on anti-peptic ulcer activity 119 Medicarpin act on DRD1 receptor. DRD1 is a kind of dopamine receptor and is widely distributed in the enteric nervous system such as colon, gastroesophageal junction, pylorus, small intestine and stomach 120. Dopamine, the substrate of DRD1, weakens human gastric motility and pressure. Thus, DA antagonists such as domperidone can improve GI mobility. Actually, DA receptors regulate gastric smooth muscle cell response through two actions: relaxation of the longitudinal smooth muscle layer and/or contraction of the circular smooth muscle layer 121. Naringenin increase the factors related to gastrointestinal movement, ICC markers (c-Kit and SCF) and AQP3 122. It alleviates dyspeptic symptoms through increasing gastrointestinal motility 123. Especially, since naringenin has the highest CI (98.94%), JGT suggested that naringenin induces an increase in gastrointestinal movement factors, ICC markers (c-kit and SCF) and AQP3 to increase gastrointestinal motility in relieving FD. It is thought to play a key role in alleviating FD (Figure 6).

This study through network pharmacological analysis would provide to the following improvements in herbal medicine treatments: (i) Evaluation of the therapeutic efficacy of JGT digestive activity against FD; and (ii) Comprehensive exploration of various pharmacological perspectives on the system-level mechanisms of herbal medicine digestive characteristics.

In conclusion, we researched the pharmacological characteristics of JGT from a system perspective, a widely prescribed herbal medicine for alleviate FD. JGT was analyzed based on a network pharmacological approach to find 84 active chemical compounds and their 84 FD-related targets related to the digestion effect of JGT. Targets of JGT were related with the regulation of biological functions such as cellular behaviors of muscle and cytokine, calcium ion concentration and homeostasis, calcium- and cytokine-mediated signalings, drug, inflammatory response, neuronal cells, oxidative stress and response to chemical, indicating system-level treatment mechanism of JGT therapies. Furthermore, through enrichment analysis, we found that the targets of JGT play a role in various pathways related to the pathophysiology of digestion, such as Apoptosis, MAPK, TNF, PI3K-Akt, Toll-like receptor signaling pathway and NOD-like receptor. Totally, a novel systematic approach to the poly-pharmacological properties of JGT was provided, and the mechanistic basis for the clinical significance of JGT in the treatment of FD was confirmed.

## Supplementary Material

Supplementary tables.
