# Supplementary material for: A study of the therapeutic mechanism of Jakyakgamcho-Tang about functional dyspepsia through network pharmacology research
Source: Int J Med Sci. 2022 Oct 17;19(13):1824–34. doi: 10.7150/ijms.77451 (PMC9682510; doi:10.7150/ijms.77451)
Supplement: Supplementary file 1 — Supplementary tables. [file ijmsv19p1824s1.pdf]

Supplementary Tables

Supplementary Table S1. List of the total chemical compounds contained in Jakyakgamcho-Tang.

Herbal medicines	Chemical compounds	OB(%)	Caco-2	DL
Paeoniae Radix Alba	(-)-alpha-cedrene	55.56	1.81	0.10
Paeoniae Radix Alba	()-trans-Myrtanol	49.66	1.17	0.06
Paeoniae Radix Alba	(+)-catechin	54.83	-0.03	0.24
Paeoniae Radix Alba	(1R)-()-Nopinone	57.86	1.23	0.05
Paeoniae Radix Alba	(3aR,6S,7aR)-6-hydroxy-6-methyl-3-methylene-3a,4,7,7a-tetrahydrobenzofuran-2,5-dione	97.79	-0.01	0.08
Paeoniae Radix Alba	(3R,3aR,6S,7aR)-6-hydroxy-3,6-dimethyl-3a,4,7,7a-tetrahydro-3H-benzofuran-2,5-dione	104.94	-0.08	0.08
Paeoniae Radix Alba	(3S,3aR,5S,6S,7aR)-5,6-dihydroxy-3,6-dimethyl-3,3a,4,5,7,7a-hexahydrobenzofuran-2-one	96.64	-0.16	0.07
Paeoniae Radix Alba	(3S,5R,8R,9R,10S,14S)-3,17-dihydroxy-4,4,8,10,14-pentamethyl-2,3,5,6,7,9-hexahydro-1H-cyclopenta[a]phenanthrene-15,16-dione	43.56	0.00	0.53
Paeoniae Radix Alba	(6R,10R)-6,10,14-trimethylpentadecan-2-one	23.30	1.41	0.10
Paeoniae Radix Alba	(Z)-(1S,5R)-beta-pinen-10-yl-beta-vicianoside	5.74	-1.56	0.67
Paeoniae Radix Alba	(Z)-(1S,5R)-beta-pinen-10-yl-beta-vicianoside_qt	50.32	1.52	0.06
Paeoniae Radix Alba	[(3S,3aR,6S,7aR)-6-hydroxy-6-methyl-2,5-dioxo-3a,4,7,7a-tetrahydro-3H-benzofuran-3-yl]methyl benzoate	17.84	-0.17	0.30
Paeoniae Radix Alba	1,2,3,6-tetra-O-galloylglucose	3.01	-2.97	0.34
Paeoniae Radix Alba	10-Methylnonadecane	10.28	1.84	0.12
Paeoniae Radix Alba	11alpha,12alpha-epoxy-3beta-23-dihydroxy-30-norolean-20-en-28,12beta-olide	64.77	0.09	0.38
Paeoniae Radix Alba	2 - methyl - 3 - (2 - propenyl) - phenol	52.06	1.64	0.03
Paeoniae Radix Alba	2,2-dimethylcyclohexanol	82.54	1.22	0.02
Paeoniae Radix Alba	24253-30-3	74.20	1.33	0.01
Paeoniae Radix Alba	24-Methylenecycloartanol	10.40	1.42	0.79
Paeoniae Radix Alba	2-Hexyl-1-decanol	17.08	1.29	0.07
Paeoniae Radix Alba	3,4,5-trihydroxybenzoic acid	31.69	-0.09	0.04
Paeoniae Radix Alba	3β,23-dihydroxy-oleana-11,13(18)-dien-28-oic acid	21.53	0.10	0.75
Paeoniae Radix Alba	3β-hydroxy-11-oxo-olean-12-en-28-oic acid	13.49	0.21	0.74

Herbal medicines	Chemical compounds	OB(%)	Caco-2	DL
Paeoniae Radix Alba	3β-hydroxy-oleana-11,13(18)-dien-28-oic acid?	17.11	0.55	0.76
Paeoniae Radix Alba	4-Chlorobutyric acid	85.82	0.74	0.01
Paeoniae Radix Alba	9-methylenefluorene	26.87	1.95	0.09
Paeoniae Radix Alba	acetic acid	47.87	0.42	0.00
Paeoniae Radix Alba	Acetyl oxide	45.13	0.65	0.01
Paeoniae Radix Alba	albiflorin	12.09	-1.54	0.77
Paeoniae Radix Alba	albiflorin R1	21.29	-1.53	0.82
Paeoniae Radix Alba	albiflorin R1_qt	26.18	-0.46	0.34
Paeoniae Radix Alba	albiflorin_qt	66.64	-0.49	0.33
Paeoniae Radix Alba	alexandrin	20.63	-0.29	0.62
Paeoniae Radix Alba	benzoyl paeoniflorin	31.27	-0.69	0.75
Paeoniae Radix Alba	beta-sitosterol	36.91	1.32	0.75
Paeoniae Radix Alba	Bicetyl	8.03	1.96	0.46
Paeoniae Radix Alba	bicyclo[3.1.1]hept-2-ene-2-methanol, 6,6-dimethyl-	49.79	1.23	0.06
Paeoniae Radix Alba	BOX	31.55	0.54	0.02
Paeoniae Radix Alba	BU3	34.87	0.19	0.01
Paeoniae Radix Alba	Cedrol	16.23	1.35	0.12
Paeoniae Radix Alba	cis-5-Octen-1-ol	31.84	1.16	0.01
Paeoniae Radix Alba	Dibutylphenol	38.90	1.73	0.06
Paeoniae Radix Alba	Dipropyl phthalate	66.30	0.78	0.10
Paeoniae Radix Alba	Dodecanal	21.52	1.40	0.03
Paeoniae Radix Alba	EEE	45.02	1.07	0.00
Paeoniae Radix Alba	Ethylisobutyrate	83.67	1.24	0.01
Paeoniae Radix Alba	gallotannin	7.36	-5.47	0.03
Paeoniae Radix Alba	galloylpaeoniflorin	3.03	-1.77	0.42
Paeoniae Radix Alba	Hederagenol	22.42	0.10	0.74
Paeoniae Radix Alba	Henicosane	8.41	1.84	0.15

Herbal medicines	Chemical compounds	OB(%)	Caco-2	DL
Paeoniae Radix Alba	Heptadekan	8.64	1.84	0.07
Paeoniae Radix Alba	Lactiflorin	49.12	-1.13	0.80
Paeoniae Radix Alba	LFA	8.46	1.83	0.13
Paeoniae Radix Alba	Methyl linolelaidate	41.93	1.46	0.17
Paeoniae Radix Alba	Methylgallate	30.91	0.26	0.05
Paeoniae Radix Alba	myristic acid	21.18	1.07	0.07
Paeoniae Radix Alba	Octacosane	8.15	1.91	0.37
Paeoniae Radix Alba	octadec-9-ene	19.50	1.87	0.09
Paeoniae Radix Alba	oxypaeoniflorin	21.88	-1.88	0.78
Paeoniae Radix Alba	Oxypaeoniflorin	8.38	-1.62	0.78
Paeoniae Radix Alba	paeoniflorgenone	87.59	-0.09	0.37
Paeoniae Radix Alba	paeoniflorin	53.87	-1.47	0.79
Paeoniae Radix Alba	paeoniflorin_qt	68.18	-0.34	0.40
Paeoniae Radix Alba	paeonol	28.79	0.93	0.04
Paeoniae Radix Alba	paeonoside	3.47	-2.71	0.71
Paeoniae Radix Alba	PENTADECYLIC ACID	20.18	1.08	0.08
Paeoniae Radix Alba	Pentagalloylglucose	3.01	-3.08	0.21
Paeoniae Radix Alba	Pisol	18.50	1.23	0.03
Paeoniae Radix Alba	Progallin A	25.61	0.33	0.06
Paeoniae Radix Alba	propyl (2R)-2-hydroxypropanoate	25.50	0.44	0.01
Paeoniae Radix Alba	Pulchinenoside A_qt	16.91	0.12	0.77
Paeoniae Radix Alba	PYG	22.98	0.69	0.02
Paeoniae Radix Alba	salicylic acid	32.13	0.63	0.03
Paeoniae Radix Alba	Satol	27.27	1.34	0.11
Paeoniae Radix Alba	Sitogluside	20.63	-0.14	0.62
Paeoniae Radix Alba	stearic acid	17.83	1.15	0.14
Paeoniae Radix Alba	sucrose	7.17	-2.89	0.23

Herbal medicines	Chemical compounds	OB(%)	Caco-2	DL
Paeoniae Radix Alba	TRD	17.89	1.78	0.03
Paeoniae Radix Alba	ZINC02169908	23.30	1.42	0.10
PRA & Licorice	Astragalin	14.03	-1.34	0.74
PRA & Licorice	DBP	64.54	0.80	0.13
PRA & Licorice	kaempferol	41.88	0.26	0.24
PRA & Licorice	Mairin	55.38	0.73	0.78
PRA & Licorice	oleanolic acid	29.02	0.59	0.76
PRA & Licorice	sitosterol	36.91	1.32	0.75
Licorice	(-)-Medicocarpin	40.99	-0.60	0.95
Licorice	()-Menthol	59.33	1.27	0.03
Licorice	(1S,2S)-1,2-dimethylcyclopentane	41.78	1.78	0.01
Licorice	(2R)-1-[2,4-dihydroxy-5-(3-methylbut-2-enyl)phenyl]-2-hydroxy-3-[4-hydroxy-3-(3-methylbut-2-enyl)phenyl]propan-1-one	1.06	0.33	0.48
Licorice	(2R)-2-[3,4-dihydroxy-5-(3-methylbut-2-enyl)phenyl]-5,7-dihydroxy-8-(3-methylbut-2-enyl)chroman-4-one	1.21	0.51	0.63
Licorice	(2R)-7-hydroxy-2-(4-hydroxyphenyl)chroman-4-one	71.12	0.41	0.18
Licorice	(2R)-7-hydroxy-2-[4-hydroxy-3-(3-methylbut-2-enyl)phenyl]chroman-4-one	5.99	0.74	0.33
Licorice	(2S)-2-[4-hydroxy-3-(3-methylbut-2-enyl)phenyl]-8,8-dimethyl-2,3-dihydropyrano[2,3-f]chromen-4-one	31.79	1.00	0.72
Licorice	(2S)-6-(2,4-dihydroxyphenyl)-2-(2-hydroxypropan-2-yl)-4-methoxy-2,3-dihydrofuro[3,2-g]chromen-7-one	60.25	0.00	0.63
Licorice	(2S)-7-hydroxy-2-(4-hydroxyphenyl)-8-(3-methylbut-2-enyl)chroman-4-one	36.57	0.72	0.32
Licorice	(3S)-2,3-dimethylpentane	35.57	1.78	0.01
Licorice	(4S)-2,4-dimethylhexane	37.13	1.77	0.01
Licorice	(E)-1-(2,4-dihydroxyphenyl)-3-(2,2-dimethylchromen-6-yl)prop-2-en-1-one	39.62	0.66	0.35
Licorice	(E)-1-(2,4-dihydroxyphenyl)-3-[4-hydroxy-3-(3-methylbut-2-enyl)phenyl]prop-2-en-1-one	1.04	0.55	0.27
Licorice	(E)-1-[2,4-dihydroxy-3-(3-methylbut-2-enyl)phenyl]-3-(2,4-dihydroxyphenyl)prop-2-en-1-one	1.36	0.50	0.30
Licorice	(E)-1-[2,4-dihydroxy-3-(3-methylbut-2-enyl)phenyl]-3-[4-hydroxy-3-(3-methylbut-2-enyl)phenyl]prop-2-en-1-one	1.02	0.81	0.45
Licorice	(E)-1-butoxyhex-2-ene	41.72	1.50	0.02

Herbal medicines	Chemical compounds	OB(%)	Caco-2	DL
Licorice	(E)-3-[3,4-dihydroxy-5-(3-methylbut-2-enyl)phenyl]-1-(2,4-dihydroxyphenyl)prop-2-en-1-one	46.27	0.41	0.31
Licorice	(E)-dodec-2-ene	17.74	1.83	0.02
Licorice	(L)-alpha-Terpineol	48.80	1.39	0.03
Licorice	(Z)-1-(2,4-dihydroxyphenyl)-3-phenylprop-2-en-1-one	73.18	0.57	0.12
Licorice	1-(5-hydroxy-2,2-dimethylchromen-6-yl)-3-(4-hydroxyphenyl)prop-2-en-1-one	5.20	0.86	0.34
Licorice	1,3-dihydroxy-8,9-dimethoxy-6-benzofurano[3,2-c]chromenone	62.90	0.40	0.53
Licorice	1,3-dihydroxy-9-methoxy-6-benzofurano[3,2-c]chromenone	48.14	0.48	0.43
Licorice	11-deoxyglycyrrhetic acid	16.21	0.51	0.76
Licorice	12-methyltetradecanoate	17.36	1.35	0.09
Licorice	18beta-glycyrrhetinic acid	22.05	0.10	0.74
Licorice	18α-hydroxyglycyrrhetic acid	41.16	-0.29	0.71
Licorice	1-Methoxyficifolinol	14.61	1.09	0.86
Licorice	1-Methoxyphaseollidin	69.98	1.01	0.64
Licorice	2-(3,4-dihydroxyphenyl)-5,7-dihydroxy-6-(3-methylbut-2-enyl)chromone	44.15	0.48	0.41
Licorice	2,2-DIMETHYLPENTANE	55.33	1.79	0.01
Licorice	2,3-dimethylhexane	46.24	1.78	0.01
Licorice	2,6,10-trimethyl-dodecane	37.80	0.08	0.03
Licorice	2',7-Dihydroxy-4'-methoxyisoflavan-7-O-β-d-glucopyranoside	10.46	-1.02	0.73
Licorice	2-[(3R)-8,8-dimethyl-3,4-dihydro-2H-pyrano[6,5-f]chromen-3-yl]-5-methoxyphenol	36.21	1.12	0.52
Licorice	21987_FLUKA	40.92	1.84	0.04
Licorice	22β-acetylglabric acid	17.76	-0.21	0.64
Licorice	24-Hydroxy-11-deoxyglycyrrhetic acid	17.57	0.26	0.76
Licorice	24-Hydroxyglycyrrhetic acid	24.17	-0.10	0.72
Licorice	2-Caren-10-al	44.74	1.37	0.05
Licorice	2-Ethyl-p-xylene	20.60	1.89	0.02
Licorice	2-heptanone	46.56	1.31	0.01
Licorice	2-methyl-5-propyl -nonane	15.28	1.81	0.03

Herbal medicines	Chemical compounds	OB(%)	Caco-2	DL
Licorice	2-methyl-6-ethyl decane	5.50	1.81	0.03
Licorice	2-Tetradecanone	17.71	1.46	0.05
Licorice	3-(2,4-dihydroxyphenyl)-8-(1,1-dimethylprop-2-enyl)-7-hydroxy-5-methoxy-coumarin	59.62	0.40	0.43
Licorice	3-(2-hydroxy-4-methoxyphenyl)-2H-chromen-7-ol	4.66	0.89	0.21
Licorice	3-(3,4-dihydroxyphenyl)-5,7-dihydroxy-8-(3-methylbut-2-enyl)chromone	66.37	0.52	0.41
Licorice	3'(γ,γ-dimethylallyl)-kievitone	1.21	0.51	0.63
Licorice	3,22-Dihydroxy-11-oxo-delta(12)-oleanene-27-alpha-methoxycarbonyl-29-oic acid	34.32	-0.06	0.55
Licorice	3,3-Dimethylpentane	41.97	1.75	0.01
Licorice	3,4,3',4'-Tetrahydroxy-2-methoxychalcone	1.33	0.64	0.20
Licorice	3-[4,6-dihydroxy-2-methoxy-3-(3-methylbut-2-enyl)phenyl]-7-hydroxy-chromone	2.47	0.43	0.44
Licorice	3-Ethylpentane	35.74	1.79	0.01
Licorice	3'-Hydroxy-4'-O-Methylglabridin	43.71	1.00	0.57
Licorice	3-Hydroxyglabrol	4.73	0.48	0.58
Licorice	3'-Methoxyglabridin	46.16	0.94	0.57
Licorice	3-methylheptane	36.61	1.79	0.01
Licorice	3-methylhexane	38.19	1.78	0.01
Licorice	3-Methylpentane	35.77	1.76	0.00
Licorice	3β-formylglabrolide	16.36	0.26	0.55
Licorice	4,2',4',alpha-Tetrahydroxydihydrochalcone	2.45	0.10	0.16
Licorice	4H-1-Benzopyran-4-one, 2-(4-(beta-D-glucopyranosyloxy)phenyl)-2,3-dihydro-5,7-dihydroxy-, (2S)-	14.03	-1.13	0.78
Licorice	5,6,7,8-Tetrahydro-2,4-dimethylquinoline	49.77	1.64	0.05
Licorice	5,6,7,8-Tetrahydro-4-methylquinoline	59.18	1.63	0.04
Licorice	5,7-dihydroxy-3-(2-hydroxy-4-methoxy-phenyl)-6-(3-methylbut-2-enyl)chromone	2.47	0.58	0.45
Licorice	5,7-dihydroxy-3-(4-methoxyphenyl)-8-(3-methylbut-2-enyl)chromone	30.49	0.90	0.41
Licorice	6″-O-acetylliquiritin	6.26	-0.48	0.82
Licorice	6-prenylated eriodictyol	39.22	0.40	0.41
Licorice	7,2',4'-trihydroxy - 5-methoxy-3 - arylcoumarin	83.71	0.24	0.27

Herbal medicines	Chemical compounds	OB(%)	Caco-2	DL
Licorice	7,4'-Dihydroxyflavone	19.18	0.56	0.18
Licorice	7-Acetoxy-2-methylisoflavone	38.92	0.74	0.26
Licorice	7-hydroxy-2-[4-hydroxy-3-(3-methylbut-2-enyl)phenyl]-6-(3-methylbut-2-enyl)chromone	4.44	0.88	0.56
Licorice	7-hydroxy-2-methyl-3-phenyl-chromone	25.80	1.00	0.18
Licorice	7-Methoxy-2-methyl isoflavone	42.56	1.16	0.20
Licorice	8-(6-hydroxy-2-benzofuranyl)-2,2-dimethyl-5-chromenol	58.44	1.00	0.38
Licorice	8-prenylated eriodictyol	53.79	0.43	0.40
Licorice	8-Prenylwighteone	23.22	0.93	0.54
Licorice	anethole	32.49	1.75	0.03
Licorice	apioglycyrrhizin	17.80	-1.91	0.14
Licorice	apioglycyrrhizin_qt	23.73	0.10	0.74
Licorice	Araboglycyrrhizin	17.73	-2.46	0.14
Licorice	Araboglycyrrhizin_qt	17.71	0.11	0.74
Licorice	Arachic acid	16.66	1.18	0.19
Licorice	Artonin E	11.38	0.34	0.80
Licorice	beta-Glycyrrhetinic acid	17.41	0.19	0.74
Licorice	beta-Terpinene	42.29	1.85	0.02
Licorice	BuOH	22.02	0.94	0.00
Licorice	butylated hydroxytoluene	40.02	1.75	0.07
Licorice	Calycosin	47.75	0.52	0.24
Licorice	Castanin	23.54	0.77	0.27
Licorice	Corylifolinin	1.04	0.81	0.27
Licorice	Cyclobutanol, 1-ethyl-	93.23	1.13	0.02
Licorice	Daidzein dimethyl ether	24.29	0.98	0.24
Licorice	dehydroglyasperins C	53.82	0.68	0.37
Licorice	DFV	32.76	0.51	0.18
Licorice	DIBP	49.63	0.85	0.13

Herbal medicines	Chemical compounds	OB(%)	Caco-2	DL
Licorice	Docosyl caffeate	3.14	1.01	0.59
Licorice	EB	49.38	1.83	0.01
Licorice	echinatin	66.58	0.38	0.17
Licorice	euchrenone	30.29	1.09	0.57
Licorice	Eurycarpin A	43.28	0.43	0.37
Licorice	formononetin	69.67	0.78	0.21
Licorice	gadelaidic acid	30.70	1.20	0.20
Licorice	Gancaonin A	51.08	0.80	0.40
Licorice	Gancaonin B	48.79	0.58	0.45
Licorice	Gancaonin C	2.87	0.14	0.42
Licorice	Gancaonin D	2.72	-0.11	0.51
Licorice	Gancaonin G	60.44	0.78	0.39
Licorice	Gancaonin H	50.10	0.60	0.78
Licorice	Gancaonin I	21.90	0.93	0.39
Licorice	Gancaonin P	1.41	0.27	0.45
Licorice	Gancaonin Q	8.98	0.86	0.60
Licorice	Gancaonin R	1.26	1.03	0.37
Licorice	Gancaonin S	1.26	0.98	0.38
Licorice	gancaonin T	1.04	0.50	0.53
Licorice	Gancaonin U	14.53	1.08	0.53
Licorice	Gancaonin V	1.24	0.73	0.34
Licorice	Glabranin	52.90	0.97	0.31
Licorice	Glabrene	46.27	0.99	0.44
Licorice	Glabridin	53.25	0.97	0.47
Licorice	glabrol	4.25	0.84	0.54
Licorice	glabrolide	17.46	0.29	0.61
Licorice	Glabrone	52.51	0.59	0.50

Herbal medicines	Chemical compounds	OB(%)	Caco-2	DL
Licorice	Glepidotin A	44.72	0.79	0.35
Licorice	Glepidotin B	64.46	0.46	0.34
Licorice	glucuronic acid	46.18	-2.05	0.06
Licorice	Glyasperin A	2.46	0.60	0.63
Licorice	glyasperin B	65.22	0.47	0.44
Licorice	Glyasperin C	45.56	0.71	0.40
Licorice	glyasperin E	4.12	0.69	0.75
Licorice	glyasperin F	75.84	0.43	0.54
Licorice	glyasperins D	29.91	0.89	0.43
Licorice	Glyasperins K	10.15	0.74	0.44
Licorice	Glyasperins M	72.67	0.49	0.59
Licorice	glyasperins Z	4.17	1.09	0.36
Licorice	Glycycoumarin	23.56	0.52	0.44
Licorice	Glycyram	19.62	-2.66	0.11
Licorice	Glycyrin	52.61	0.59	0.47
Licorice	Glycyrol	90.78	0.71	0.67
Licorice	glycyroside	37.25	-1.58	0.79
Licorice	glycyrrhetol	14.66	0.36	0.75
Licorice	Glycyrrhiza flavonol A	41.28	-0.09	0.60
Licorice	glycyrrhizin	9.06	-2.23	0.11
Licorice	glyinflanin A	1.06	0.69	0.48
Licorice	Glypallichalcone	61.60	0.76	0.19
Licorice	Glyzaglabrin	61.07	0.34	0.35
Licorice	Heptan	41.80	1.77	0.00
Licorice	HEX	52.50	1.78	0.00
Licorice	Hirsutrin	1.86	-1.66	0.77
Licorice	Hispaglabridin A	14.60	1.12	0.73

Herbal medicines	Chemical compounds	OB(%)	Caco-2	DL
Licorice	Hispaglabridin B	22.94	1.18	0.88
Licorice	HMO	38.37	0.79	0.21
Licorice	ICO	33.86	0.82	0.05
Licorice	icos-5-enoic acid	30.70	1.22	0.20
Licorice	Inermine	75.18	0.89	0.54
Licorice	Inflacoumarin A	39.71	0.73	0.33
Licorice	isoglabrolide	14.77	0.32	0.62
Licorice	isoglycycoumarin	22.09	0.55	0.60
Licorice	Isoglycyrol	44.70	0.91	0.84
Licorice	isograbrol	11.04	0.87	0.50
Licorice	ISOHEPTANE	59.94	1.81	0.01
Licorice	Isohexane	56.13	1.77	0.00
Licorice	Isolicoflavonol	45.17	0.54	0.42
Licorice	isoliquiritigenin	85.32	0.44	0.15
Licorice	Isoliquiritin	8.61	-1.36	0.60
Licorice	Isoononin	8.29	-1.00	0.79
Licorice	isorhamnetin	49.60	0.31	0.31
Licorice	Isoschaftoside	17.38	-2.62	0.83
Licorice	Isotrifoliol	31.94	0.53	0.42
Licorice	Isoviolanthin	18.79	-2.43	0.81
Licorice	Izoforon	44.98	1.28	0.03
Licorice	Jaranol	50.83	0.61	0.29
Licorice	Kanzonol E	5.77	0.98	0.71
Licorice	Kanzonol F	32.47	1.18	0.89
Licorice	Kanzonol H	16.92	0.96	0.80
Licorice	Kanzonol Z	21.77	0.50	0.76
Licorice	kanzonols K	0.97	0.76	0.66

Herbal medicines	Chemical compounds	OB(%)	Caco-2	DL
Licorice	kanzonols L	0.98	0.70	0.78
Licorice	kanzonols T	17.87	0.05	0.67
Licorice	kanzonols W	50.48	0.63	0.52
Licorice	kanzonols X	7.56	1.10	0.56
Licorice	Karenzu DK2	62.26	0.94	0.10
Licorice	Licoagrocarpin	58.81	1.23	0.58
Licorice	Licoagroisoflavone	57.28	0.71	0.49
Licorice	licoagropin	27.14	1.63	0.51
Licorice	licochalcone a	40.79	0.82	0.29
Licorice	Licochalcone B	76.76	0.47	0.19
Licorice	licochalcone C	4.44	0.63	0.29
Licorice	licochalcone G	49.25	0.64	0.32
Licorice	licochalconeD	1.01	0.47	0.34
Licorice	Licocoumarone	33.21	0.84	0.36
Licorice	Licoflavone	18.75	0.82	0.33
Licorice	Licoflavonol	8.75	0.49	0.40
Licorice	licoisoflavanone	52.47	0.39	0.54
Licorice	Licoisoflavone	41.61	0.37	0.42
Licorice	Licoisoflavone B	38.93	0.46	0.55
Licorice	Liconeolignan	4.41	1.00	0.40
Licorice	licopyranocoumarin	80.36	0.13	0.65
Licorice	Licorice glycoside A	5.95	-2.37	0.35
Licorice	licorice glycoside E	32.89	-2.06	0.27
Licorice	licorice-saponin B2	58.55	-2.40	0.11
Licorice	licorice-saponin C2	59.66	-2.28	0.11
Licorice	licorice-saponin C2_qt	17.33	0.48	0.76
Licorice	licorice-saponin F3	17.68	-2.82	0.03

Herbal medicines	Chemical compounds	OB(%)	Caco-2	DL
Licorice	licorice-saponin F3_qt	27.53	0.74	0.64
Licorice	licorice-saponin G2	6.39	-2.01	0.11
Licorice	licorice-saponin G2_qt	22.78	-0.27	0.72
Licorice	licorice-saponin H2	44.37	-2.08	0.11
Licorice	licorice-saponin H2_qt	22.91	0.01	0.74
Licorice	licorice-saponin J2	6.25	-2.25	0.11
Licorice	licorice-saponin J2_qt	28.30	0.04	0.74
Licorice	licorice-saponin K2	7.82	-2.64	0.11
Licorice	licorice-saponin K2_qt	27.79	0.05	0.75
Licorice	Licoricidin	0.99	0.96	0.62
Licorice	Licoricone	63.58	0.53	0.47
Licorice	Licoriisoflavan A	3.68	1.10	0.66
Licorice	licuraside	5.25	-1.92	0.77
Licorice	liquiritin	65.69	-1.06	0.74
Licorice	Liquiritin apioside	29.23	-1.88	0.82
Licorice	liquoric acid	25.44	-0.01	0.55
Licorice	Lupiwighteone	51.64	0.68	0.37
Licorice	Medicarpin	49.22	1.00	0.34
Licorice	Methylcyclopentane	55.78	1.79	0.01
Licorice	Methylheptane	28.65	1.79	0.01
Licorice	Mipax	57.40	0.64	0.06
Licorice	Morusin	11.52	0.51	0.76
Licorice	m-xylene	47.43	1.83	0.01
Licorice	Narcissoside	5.09	-2.14	0.65
Licorice	naringenin	59.29	0.28	0.21
Licorice	naringin	6.92	-1.99	0.78
Licorice	neoisoliquiritin	21.18	-1.41	0.58

Herbal medicines	Chemical compounds	OB(%)	Caco-2	DL
Licorice	neoliquiritin	13.01	-1.08	0.71
Licorice	Neouralenol	12.76	0.24	0.46
Licorice	nicotiflorin	3.64	-1.77	0.73
Licorice	Nortangeretin	17.90	0.24	0.27
Licorice	OCT	29.72	1.78	0.01
Licorice	Octadiene	34.53	1.81	0.01
Licorice	Odoratin	49.95	0.42	0.30
Licorice	Ononin	11.52	-0.74	0.78
Licorice	o-xylene	45.55	1.85	0.01
Licorice	Pentadecanol	13.73	1.30	0.06
Licorice	PENTYLFURAN	54.59	1.72	0.02
Licorice	Phaseol	78.77	0.76	0.58
Licorice	Phaseolinisoflavan	32.01	1.01	0.45
Licorice	Pinocembrin	64.72	0.61	0.18
Licorice	protocatechuic acid	25.37	0.10	0.04
Licorice	Prunetin	5.41	0.65	0.24
Licorice	p-xylene	48.74	1.83	0.01
Licorice	quercetin	46.43	0.05	0.28
Licorice	Quercetin der.	46.45	0.39	0.33
Licorice	rutin	3.20	-1.93	0.68
Licorice	schaftoside	7.88	-2.46	0.75
Licorice	Scopoletol	27.77	0.71	0.08
Licorice	Semilicoisoflavone B	48.78	0.45	0.55
Licorice	Sextone B	56.20	1.77	0.01
Licorice	shinpterocarpin	80.30	1.10	0.73
Licorice	Sigmoidin-B	34.88	0.42	0.41
Licorice	Uralene	11.70	0.63	0.49

Herbal medicines	Chemical compounds	OB(%)	Caco-2	DL
Licorice	uralenneoside	24.96	-1.01	0.17
Licorice	Uralenol	8.55	0.35	0.46
Licorice	Uralenol-3-methylether	1.41	0.39	0.49
Licorice	uralsaponin B	7.92	-2.60	0.11
Licorice	ursolic acid	16.77	0.67	0.75
Licorice	Vestitol	74.66	0.86	0.21
Licorice	Vicenin-2	3.42	-3.15	0.78
Licorice	violanthin	4.17	-2.01	0.81
Licorice	vitexin	3.05	-1.52	0.71
Licorice	WLN: 4OVR	48.41	1.31	0.04
Licorice	WLN: VH6	19.59	1.29	0.01
Licorice	Xambioona	54.85	1.09	0.87
Licorice	Yinyanghuo D	13.99	0.61	0.38
Licorice	α-cubebol	64.81	1.32	0.09

OB, oral bioavailability; Caco-2, Caco-2 cell permeability; DL, drug-likeness score.

Supplementary Tables

Supplementary Table S2. List of the active chemical compounds in Jakyakgamcho-Tang.

Herbal medicines	Chemical compounds	OB(%)	Caco-2	DL
Paeoniae Radix Alba	(+)-catechin	54.83	-0.03	0.24
Paeoniae Radix Alba	(3S,5R,8R,9R,10S,14S)-3,17-dihydroxy-4,4,8,10,14-pentamethyl-2,3,5,6,7,9-hexahydro-1H-cyclopenta[a]phenanthrene-15,16-dione	43.56	0.00	0.53
Paeoniae Radix Alba	11alpha,12alpha-epoxy-3beta-23-dihydroxy-30-norolean-20-en-28,12beta-olide	64.77	0.09	0.38
Paeoniae Radix Alba	beta-sitosterol	36.91	1.32	0.75
Paeoniae Radix Alba	paeoniflorgenone	87.59	-0.09	0.37
Paeoniae Radix Alba	paeoniflorin_qt	68.18	-0.34	0.40
PRA & Licorice	kaempferol	41.88	0.26	0.24
PRA & Licorice	Mairin	55.38	0.73	0.78
PRA & Licorice	sitosterol	36.91	1.32	0.75
Licorice	(2R)-7-hydroxy-2-(4-hydroxyphenyl)chroman-4-one	71.12	0.41	0.18
Licorice	(2S)-2-[4-hydroxy-3-(3-methylbut-2-enyl)phenyl]-8,8-dimethyl-2,3-dihydropyrano[2,3-f]chromen-4-one	31.79	1.00	0.72
Licorice	(2S)-6-(2,4-dihydroxyphenyl)-2-(2-hydroxypropan-2-yl)-4-methoxy-2,3-dihydrofuro[3,2-g]chromen-7-one	60.25	0.00	0.63
Licorice	(2S)-7-hydroxy-2-(4-hydroxyphenyl)-8-(3-methylbut-2-enyl)chroman-4-one	36.57	0.72	0.32
Licorice	(E)-1-(2,4-dihydroxyphenyl)-3-(2,2-dimethylchromen-6-yl)prop-2-en-1-one	39.62	0.66	0.35
Licorice	(E)-3-[3,4-dihydroxy-5-(3-methylbut-2-enyl)phenyl]-1-(2,4-dihydroxyphenyl)prop-2-en-1-one	46.27	0.41	0.31
Licorice	1,3-dihydroxy-8,9-dimethoxy-6-benzofurano[3,2-c]chromenone	62.90	0.40	0.53
Licorice	1,3-dihydroxy-9-methoxy-6-benzofurano[3,2-c]chromenone	48.14	0.48	0.43
Licorice	18α-hydroxyglycyrrhetic acid	41.16	-0.29	0.71
Licorice	1-Methoxyphaseollidin	69.98	1.01	0.64
Licorice	2-(3,4-dihydroxyphenyl)-5,7-dihydroxy-6-(3-methylbut-2-enyl)chromone	44.15	0.48	0.41
Licorice	2-[(3R)-8,8-dimethyl-3,4-dihydro-2H-pyrano[6,5-f]chromen-3-yl]-5-methoxyphenol	36.21	1.12	0.52
Licorice	3-(2,4-dihydroxyphenyl)-8-(1,1-dimethylprop-2-enyl)-7-hydroxy-5-methoxy-coumarin	59.62	0.40	0.43
Licorice	3-(3,4-dihydroxyphenyl)-5,7-dihydroxy-8-(3-methylbut-2-enyl)chromone	66.37	0.52	0.41

Herbal medicines	Chemical compounds	OB(%)	Caco-2	DL
Licorice	3,22-Dihydroxy-11-oxo-delta(12)-oleanene-27-alpha-methoxycarbonyl-29-oic acid	34.32	-0.06	0.55
Licorice	3'-Hydroxy-4'-O-Methylglabridin	43.71	1.00	0.57
Licorice	3'-Methoxyglabridin	46.16	0.94	0.57
Licorice	5,7-dihydroxy-3-(4-methoxyphenyl)-8-(3-methylbut-2-enyl)chromone	30.49	0.90	0.41
Licorice	6-prenylated eriodictyol	39.22	0.40	0.41
Licorice	7,2',4'-trihydroxy - 5-methoxy-3 - arylcoumarin	83.71	0.24	0.27
Licorice	7-Acetoxy-2-methylisoflavone	38.92	0.74	0.26
Licorice	7-Methoxy-2-methyl isoflavone	42.56	1.16	0.20
Licorice	8-(6-hydroxy-2-benzofuranyl)-2,2-dimethyl-5-chromenol	58.44	1.00	0.38
Licorice	8-prenylated eriodictyol	53.79	0.43	0.40
Licorice	Calycosin	47.75	0.52	0.24
Licorice	dehydroglyasperins C	53.82	0.68	0.37
Licorice	DFV	32.76	0.51	0.18
Licorice	euchrenone	30.29	1.09	0.57
Licorice	Eurycarpin A	43.28	0.43	0.37
Licorice	formononetin	69.67	0.78	0.21
Licorice	gadelaidic acid	30.70	1.20	0.20
Licorice	Gancaonin A	51.08	0.80	0.40
Licorice	Gancaonin B	48.79	0.58	0.45
Licorice	Gancaonin G	60.44	0.78	0.39
Licorice	Gancaonin H	50.10	0.60	0.78
Licorice	Glabranin	52.90	0.97	0.31
Licorice	Glabrene	46.27	0.99	0.44
Licorice	Glabridin	53.25	0.97	0.47
Licorice	Glabrone	52.51	0.59	0.50
Licorice	Glepidotin A	44.72	0.79	0.35
Licorice	Glepidotin B	64.46	0.46	0.34

Herbal medicines	Chemical compounds	OB(%)	Caco-2	DL
Licorice	glyasperin B	65.22	0.47	0.44
Licorice	Glyasperin C	45.56	0.71	0.40
Licorice	glyasperin F	75.84	0.43	0.54
Licorice	Glyasperins M	72.67	0.49	0.59
Licorice	Glycyrin	52.61	0.59	0.47
Licorice	Glycyrol	90.78	0.71	0.67
Licorice	Glycyrrhiza flavonol A	41.28	-0.09	0.60
Licorice	Glypallichalcone	61.60	0.76	0.19
Licorice	Glyzaglabrin	61.07	0.34	0.35
Licorice	HMO	38.37	0.79	0.21
Licorice	icos-5-enoic acid	30.70	1.22	0.20
Licorice	Inermine	75.18	0.89	0.54
Licorice	Inflacoumarin A	39.71	0.73	0.33
Licorice	Isoglycyrol	44.70	0.91	0.84
Licorice	Isolicoflavonol	45.17	0.54	0.42
Licorice	isorhamnetin	49.60	0.31	0.31
Licorice	Isotrifoliol	31.94	0.53	0.42
Licorice	Jaranol	50.83	0.61	0.29
Licorice	Kanzonol F	32.47	1.18	0.89
Licorice	kanzonols W	50.48	0.63	0.52
Licorice	Licoagrocarpin	58.81	1.23	0.58
Licorice	Licoagroisoflavone	57.28	0.71	0.49
Licorice	licochalcone a	40.79	0.82	0.29
Licorice	Licochalcone B	76.76	0.47	0.19
Licorice	licochalcone G	49.25	0.64	0.32
Licorice	Licocoumarone	33.21	0.84	0.36
Licorice	licoisoflavanone	52.47	0.39	0.54

Herbal medicines	Chemical compounds	OB(%)	Caco-2	DL
Licorice	Licoisoflavone	41.61	0.37	0.42
Licorice	Licoisoflavone B	38.93	0.46	0.55
Licorice	licopyranocoumarin	80.36	0.13	0.65
Licorice	Licoricone	63.58	0.53	0.47
Licorice	Lupiwighteone	51.64	0.68	0.37
Licorice	Medicarpin	49.22	1.00	0.34
Licorice	naringenin	59.29	0.28	0.21
Licorice	Odoratin	49.95	0.42	0.30
Licorice	Phaseol	78.77	0.76	0.58
Licorice	Phaseolinisoflavan	32.01	1.01	0.45
Licorice	quercetin	46.43	0.05	0.28
Licorice	Quercetin der.	46.45	0.39	0.33
Licorice	Semilicoisoflavone B	48.78	0.45	0.55
Licorice	shinpterocarpin	80.30	1.10	0.73
Licorice	Sigmoidin-B	34.88	0.42	0.41
Licorice	Vestitol	74.66	0.86	0.21
Licorice	Xambioona	54.85	1.09	0.87

OB, oral bioavailability; Caco-2, Caco-2 cell permeability; DL, drug-likeness score

Supplementary Table S3. List of the targets of active chemical compounds of Jakyakgamcho-Tang.

Herbal medicines	Chemical compounds	Targets
Paeoniae Radix Alba	(+)-catechin	ampC, CALM1, ESR1*, HAS2, HSP90AA1, NCOA2, PRKACA, PTGS1*, PTGS2*, RXRA
Paeoniae Radix Alba	(3S,5R,8R,9R,10S,14S)-3,17-dihydroxy-4,4,8,10,14-pentamethyl-2,3,5,6,7,9-hexahydro-1H-cyclopenta[a]phenanthrene-15,16-dione	NR3C2, PGR
Paeoniae Radix Alba	beta-sitosterol	ADRA1A*, ADRA1B*, ADRB2*, BAX*, BCL2*, camC, CASP3*, CASP8, CASP9, CHRM1, CHRM2, CHRM3*, CHRM4, CHRNA2, CHRNA7, DRD1, GABRA1, GABRA2, GABRA3, GABRA5, HSP90AA1, HTR2A*, JUN*, KCNH2*, MAP2, NCOA2, OPRM1*, PDE3A, PGR, PIK3CG*, PON1*, PRKACA, PRKCA, PTGS1*, PTGS2*, SCN5A*, SLC6A4*, TGFB1*
Paeoniae Radix Alba	paeoniflorgenone	GABRA1
PRA&Licorice	kaempferol	ACHE*, ADRA1B*, AHR, AHSA1, AKR1C3, AKT1*, ALOX5, AR, BAX*, BCL2*, CALM1, CASP3*, CDK1, CHRM1, CHRM2, CYP1A1*, CYP1B1, CYP3A4*, DIO1, DPP4*, DPP4*, F2*, F7, GABRA1, GABRA2, GSTM1*, GSTM2, HAS2, HSP90AA1, ICAM1, IKBKB*, INSR, JUN*, MAPK8*, MMP1, NCOA2, NOS2*, NOS3*, NR1I2*, NR1I3*, pbsA1, PGR, PGR, PIK3CG*, PPARG*, PPP3CA, PRKACA, PRSS1, PRXC1A, PSMD3, PTGS1*, PTGS2*, RELA*, SELE, SLC2A4, SLC6A2*, SLPI, STAT1, TNF*, TOP2A, VCAM1, XDH
PRA&Licorice	Mairin	PGR
PRA&Licorice	sitosterol	NCOA2, NR3C2, PGR, PGR
Licorice	(2R)-7-hydroxy-2-(4-hydroxyphenyl)chroman-4-one	ADRB2*, ampC, CALM1, ESR1*, GABRA1, HSP90AA1, MAOB*, PDE3A, PIK3CG*, PKIA, PRKACA, PTGS1*, PTGS2*, RXRA, SLC6A4*
Licorice	(2S)-2-[4-hydroxy-3-(3-methylbut-2-	AR, CALM1, ESR1*, ESR2, F10, GSK3B, KCNH2*, MAPK14, NOS2*, PIM1,

Herbal medicines	Chemical compounds	Targets
	enyl)phenyl]-8,8-dimethyl-2,3-dihydropyrano[2,3-f]chromen-4-one	PPARG*, PTGS2*
Licorice	(2S)-6-(2,4-dihydroxyphenyl)-2-(2-hydroxypropan-2-yl)-4-methoxy-2,3-dihydrofuro[3,2-g]chromen-7-one	ACHE*, AR, CALM1, CCNA2, CDK2, CHEK1, DPP4*, ESR1*, ESR2, F10, F2*, F7, GSK3B, KDR, MAPK14, NOS2*, PIM1, PPARG*, PRSS1, PTGS2*, TOP2A
Licorice	(2S)-7-hydroxy-2-(4-hydroxyphenyl)-8-(3-methylbut-2-enyl)chroman-4-one	ADRA1B*, ADRB2*, CALM1, ESR1*, ESR2, F10, HSP90AA1, NOS2*, PDE3A, PTGS1*, PTGS2*, SCN5A*
Licorice	(E)-1-(2,4-dihydroxyphenyl)-3-(2,2-dimethylchromen-6-yl)prop-2-en-1-one	ADRA1B*, AR, CA2*, CALM1, CCNA2, CDK2, CHEK1, ESR1*, ESR2, F10, GSK3B, MAPK14, NCOA2, NOS2*, PIM1, PPARG*, PTGS1*, PTGS2*, RXRA, SCN5A*
Licorice	(E)-3-[3,4-dihydroxy-5-(3-methylbut-2-enyl)phenyl]-1-(2,4-dihydroxyphenyl)prop-2-en-1-one	AR, CALM1, CCNA2, CDK2, ESR1*, GSK3B, HSP90AA1, MAPK14, NCOA2, PIM1, PPARG*, PTGS2*
Licorice	1,3-dihydroxy-8,9-dimethoxy-6-benzofurano[3,2-c]chromenone	AR, CDK2, CHEK1, ESR1*, GSK3B, HSP90AA1, MAPK14, PPARG*, PRKACA
Licorice	1,3-dihydroxy-9-methoxy-6-benzofurano[3,2-c]chromenone	CCNA2, CDK2, CHEK1, ESR1*, ESR2, GSK3B, HSP90AA1, MAPK14, PPARG*, PRKACA
Licorice	1-Methoxyphaseollidin	ADRA1B*, ADRA1D*, ADRB2*, AR, CALM1, CCNA2, CDK2, ESR1*, ESR2, F10, F2*, GSK3B, HSP90AA1, KCNH2*, KDR, MAPK14, NCOA1, NCOA2, NOS2*, NOS3*, PIK3CG*, PIM1, PPARG*, PRSS1, PTGS1*, PTGS2*, RXRA, SCN5A*, TOP2A
Licorice	2-(3,4-dihydroxyphenyl)-5,7-dihydroxy-6-(3-methylbut-2-enyl)chromone	ADRB2*, AR, CALM1, CCNA2, CDK2, CHEK1, DPP4*, F10, F2*, F7, HSP90AA1, PIM1, PPARG*, PRSS1, PTGS2*, SCN5A*
Licorice	2-[(3R)-8,8-dimethyl-3,4-dihydro-2H-pyrano[6,5-f]chromen-3-yl]-5-methoxyphenol	ACHE*, ADRA1B*, ADRB2*, AR, CALM1, CCNA2, CDK2, CHEK1, CHRM1, CHRM3*, ESR1*, ESR2, F10, GSK3B, KCNH2*, KCNMA1, MAPK14, NCOA1, NCOA2, NOS2*, NOS3*, PIM1, PPARG*, PRKACA, PRSS1, PTGS1*, PTGS2*,

Herbal medicines	Chemical compounds	Targets
		RXRA, SCN5A*, SLC6A3
Licorice	3-(2,4-dihydroxyphenyl)-8-(1,1-dimethylprop-2-enyl)-7-hydroxy-5-methoxy-coumarin	AR, CALM1, CDK2, CHEK1, DPP4*, ESR1*, ESR2, F10, F2*, F7, GSK3B, HSP90AA1, KCNH2*, KDR, MAPK14, NCOA1, NCOA2, NOS2*, PIM1, PPARG*, PRSS1, PTGS2*, TOP2A
Licorice	3-(3,4-dihydroxyphenyl)-5,7-dihydroxy-8-(3-methylbut-2-enyl)chromone	AR, CALM1, CCNA2, CDK2, CHEK1, ESR1*, F10, F2*, GSK3B, HSP90AA1, MAPK14, NCOA2, NOS2*, PIM1, PPARG*, PRSS1, PTGS2*, PTPN1
Licorice	3'-Hydroxy-4'-O-Methylglabridin	ADRA1B*, ADRB2*, AR, CALM1, CCNA2, CDK2, CHEK1, ESR1*, ESR2, F10, F7, GSK3B, HSP90AA1, KCNH2*, KCNMA1, KDR, MAPK14, NCOA1, NCOA2, NOS2*, PIM1, PPARG*, PRKACA, PRSS1, PTGS1*, PTGS2*, SCN5A*, TOP2A
Licorice	3'-Methoxyglabridin	ACHE*, ADRA1B*, ADRB2*, AR, CALM1, CCNA2, CDK2, CHEK1, ESR1*, ESR2, F10, F7, GSK3B, HSP90AA1, KCNH2*, KCNMA1, MAPK14, NCOA1, NCOA2, NOS2*, PIM1, PPARG*, PRSS1, PTGS1*, PTGS2*, RXRA, SCN5A*, TOP2A
Licorice	5,7-dihydroxy-3-(4-methoxyphenyl)-8-(3-methylbut-2-enyl)chromone	AR, CALM1, CCNA2, CDK2, CHEK1, DPP4*, ESR1*, ESR2, F10, GSK3B, HSP90AA1, KCNH2*, MAPK14, NCOA2, NOS2*, PIM1, PPARG*, PRSS1, PTGS2*, TOP2A
Licorice	6-prenylated eriodictyol	CALM1, ESR1*, F10, F7, HSP90AA1, NOS2*, PTGS2*, SCN5A*
Licorice	7,2',4'-trihydroxy - 5-methoxy-3 - arylcoumarin	AR, CDK2, CHEK1, DPP4*, ESR1*, ESR2, GSK3B, HSP90AA1, MAPK14, NOS2*, PIM1, PPARG*, PRKACA, PTGS1*, PTGS2*
Licorice	7-Acetoxy-2-methylisoflavone	ACHE*, ADRA1B*, ADRA1D*, ADRB2*, AR, CALM1, CDK2, CHEK1, DPP4*, ESR1*, F2*, GABRA1, GSK3B, HSP90AA1, MAPK14, NCOA2, NOS2*, NOS3*, PDE3A, PPARG*, PRSS1, PTGS1*, PTGS2*, RXRA, SCN5A*
Licorice	7-Methoxy-2-methyl isoflavone	ACHE*, ADRA1B*, ADRA1D*, ADRB1*, ADRB2*, AR, CALM1, CCNA2, CDK2, CHEK1, CHRM1, CHRM3*, CHRM5, CHRNA7, DPP4*, DRD1, ESR1*, ESR2, F2*, GABRA1, MAPK14, NCOA1, GSK3B, HSP90AA1, IGHG1, LTA4H,

Herbal medicines	Chemical compounds	Targets
		MAOB*, NCOA2, NOS2*, NOS3*, OPRM1*, PDE3A, PIM1, PKIA, PPARG*, PRKACA, PRSS1, PTGS1*, PTGS2*, RXRA, SCN5A*, SLC6A3, SLC6A4*
Licorice	8-(6-hydroxy-2-benzofuranyl)-2,2-dimethyl-5-chromenol	ESR1*, HSP90AA1, NOS2*, PIK3CG*, PTGS2*, RXRA
Licorice	8-prenylated eriodictyol	CALM1, ESR1*, F10, F7, HSP90AA1, NCOA1, PTGS2*, SCN5A*
Licorice	Calycosin	ADRB2*, AR, CALM1, CCNA2, CDK2, CHEK1, DPP4*, ESR1*, ESR2, GSK3B, HSP90AA1, MAPK14, NCOA2, NOS2*, PDE3A, PIM1, PPARG*, PRKACA, PRSS1, PTGS1*, PTGS2*, RXRA
Licorice	dehydroglyasperins C	ADRB2*, AR, CALM1, CCNA2, CDK2, CHEK1, ESR1*, ESR2, F10, HSP90AA1, MAPK14, NCOA2, NOS2*, PIM1, PPARG*, PRSS1, PTGS2*, SCN5A*
Licorice	DFV	ADRB2*, ampC, ESR1*, HSP90AA1, MAOB*, PIK3CG*, PKIA, PRKACA, PTGS1*, PTGS2*, RXRA, SLC6A4*
Licorice	euchrenone	BACE1, CALM1, ESR1*, ESR2, F10, KCNH2*, NOS2*, PIM1, PTGS2*, SCN5A*
Licorice	Eurycarpin A	AR, CALM1, CCNA2, CDK2, CHEK1, DPP4*, ESR1*, ESR2, F10, F2*, GSK3B, HSP90AA1, MAPK14, NOS2*, PIM1, PPARG*, PRSS1, PTGS2*, SCN5A*
Licorice	formononetin	ACHE*, ADRA1A*, ADRB2*, ampC, AR, ATP5B, CALM1, CCNA2, CDK2, CHEK1, CHRM1, DPP4*, ESR1*, ESR2, F2*, GSK3B, HSD3B1, HSD3B2, HSP90AA1, IL4*, JUN*, MAOB*, MAPK14, MT-ND6, NOS2*, NOS3*, PDE3A, PIM1, PKIA, PPARG*, PRKACA, PRSS1, PTGS1*, PTGS2*, RXRA, SIRT1, SLC6A3, SLC6A4*
Licorice	gadelaidic acid	NCOA2
Licorice	Gancaonin A	ACHE*, AR, CALM1, CCNA2, CHEK1, DPP4*, ESR1*, ESR2, F10, F2*, GSK3B, HSP90AA1, NCOA2, NOS2*, PIM1, PPARG*, PRSS1, PTGS2*, SCN5A*, TOP2A
Licorice	Gancaonin B	ADRA1B*, ADRB2*, AR, CALM1, CCNA2, CHEK1, DPP4*, ESR1*, ESR2, F10,

Herbal medicines	Chemical compounds	Targets
		F2*, F7, GSK3B, HSP90AA1, KDR, NCOA2, NOS2*, PIM1, PPARG*, PRSS1, PTGS2*, TOP2A
Licorice	Gancaonin G	AR, CALM1, CCNA2, CHEK1, DPP4*, ESR1*, ESR2, F10, F2*, GSK3B, HSP90AA1, MAPK14, NCOA2, NOS2*, NOS3*, PIM1, PPARG*, PRSS1, PTGS2*, TOP2A
Licorice	Gancaonin H	AR, CALM1, CCNA2, ESR1*, F10, HSP90AA1, KDR, NCOA2, PIM1, PRSS1, PTGS2*, TOP2A
Licorice	Glabranin	CALM1, ESR1*, F10, HSP90AA1, NOS2*, NOS3*, PDE3A, PRKACA, PTGS1*, PTGS2*, SCN5A*
Licorice	Glabrene	ADRB2*, AR, CALM1, CDK2, ESR1*, ESR2, F10, GSK3B, HSP90AA1, MAPK14, NCOA2, NOS2*, PIM1, PPARG*, PRSS1, PTGS1*, PTGS2*, RXRA, SCN5A*
Licorice	Glabridin	ACHE*, ADRA1B*, ADRB2*, AR, CALM1, CCNA2, CDK2, CHEK1, CHRM1, ESR1*, ESR2, GSK3B, IGHG1, MAPK14, NCOA1, NCOA2, NOS2*, PIM1, PPARG*, PRKACA, PRSS1, PTGS2*, RXRA, SCN5A*
Licorice	Glabrone	ACHE*, AR, CALM1, CCNA2, CDK2, CHEK1, DPP4*, ESR1*, ESR2, F10, F2*, GSK3B, MAPK14, NOS2*, PIM1, PPARG*, PRSS1, PTGS1*, PTGS2*, RXRA, SCN5A*
Licorice	Glepidotin A	AR, CALM1, CCNA2, CDK2, CHEK1, DPP4*, ESR1*, F10, F2*, F7, GSK3B, HSP90AA1, IGHG1, KDR, MAPK14, NOS2*, NOS3*, PDE3A, PIM1, PPARG*, PRSS1, PTGS1*, PTGS2*, RXRA, SCN5A*, TOP2A
Licorice	Glepidotin B	ADRA1B*, CALM1, ESR1*, F10, F7, HSP90AA1, IGHG1, NCOA1, NOS3*, PDE3A, PTGS1*, PTGS2*, RXRA, SCN5A*, TOP2A
Licorice	glyasperin B	ACHE*, AR, CALM1, CCNA2, CDK2, DPP4*, ESR1*, ESR2, F10, F2*, F7, GSK3B, HSP90AA1, KDR, NCOA2, NOS2*, PIM1, PPARG*, PRSS1, PTGS2*, TOP2A

Herbal medicines	Chemical compounds	Targets
Licorice	Glyasperin C	ACHE*, AR, CALM1, CCNA2, CDK2, CHEK1, DPP4*, ESR1*, ESR2, F10, F2*, GSK3B, HSP90AA1, KCNH2*, MAPK14, NCOA2, NOS2*, PIM1, PPARG*, PRSS1, PTGS2*, RXRA, SCN5A*, TOP2A
Licorice	glyasperin F	AR, CALM1, CCNA2, CDK2, ESR1*, ESR2, F10, GSK3B, HSP90AA1, MAPK14, NOS2*, PIM1, PPARG*, PRSS1, PTGS1*, PTGS2*, SCN5A*, TOP2A
Licorice	Glyasperins M	ACHE*, AR, CALM1, CCNA2, CDK2, ESR1*, ESR2, F10, F7, GSK3B, HSP90AA1, KCNH2*, KCNMA1, KDR, NCOA1, NCOA2, NOS2*, PIM1, PPARD, PPARG*, PRKACA, PRSS1, PTGS1*, PTGS2*, SCN5A*, TOP2A
Licorice	Glycyrin	AR, CALM1, CHEK1, DPP4*, ESR1*, ESR2, F10, F2*, KCNH2*, KDR, NCOA2, NOS2*, PIM1, PPARG*, PRSS1, PTGS2*, TOP2A
Licorice	Glycyrol	CCNA2, CHEK1, ESR1*, F2*, GSK3B, KDR, MAPK14, NOS2*, PIM1, PPARG*, PTGS2*
Licorice	Glycyrrhizaflavonol A	ACHE*, AR, CALM1, CCNA2, CDK2, DPP4*, ESR1*, ESR2, F10, F7, GSK3B, HSP90AA1, NOS2*, PIM1, PRSS1, PTGS2*, TOP2A
Licorice	Glypallichalcone	ADRA1B*, ADRB2*, AR, CA2*, CALM1, CCNA2, CDK2, CHEK1, CHRM1, ESR1*, ESR2, GSK3B, HSP90AA1, LTA4H, MAOB*, MAPK14, NCOA1, NOS2*, PDE3A, PKIA, PPARG*, PRKACA, PTGS1*, PTGS2*, SCN5A*, SLC6A3, SLC6A4*
Licorice	Glyzaglabrin	AR, CCNA2, CDK2, CHEK1, DPP4*, ESR1*, ESR2, GSK3B, HSP90AA1, MAPK14, NOS2*, PIK3CG*, PIM1, PPARG*, PRKACA, PRSS1, PTGS1*, PTGS2*
Licorice	HMO	ADRB2*, AR, CALM1, CCNA2, CDK2, CHEK1, CHRM1, DPP4*, ESR1*, ESR2, GSK3B, IGHG1, MAOB*, MAPK14, NOS2*, PDE3A, PIM1, PKIA, PPARG*, PRKACA, PRSS1, PTGS1*, PTGS2*, RXRA, SCN5A*, SLC6A3, SLC6A4*
Licorice	icos-5-enoic acid	NCOA2
Licorice	Inermine	ADRA1B*, ADRA1D*, ADRB2*, CALM1, CHRM1, CHRM3*, HSP90AA1,

Herbal medicines	Chemical compounds	Targets
		HTR3A*, IGHG1, OPRM1*, PIK3CG*, PRKACA, PRSS1, PTGS1*, PTGS2*, RXRA, SCN5A*
Licorice	Inflacoumarin A	ADRB2*, AR, CALM1, DPP4*, ESR1*, F10, F2*, HSP90AA1, NCOA2, PIM1, PPARG*, PRSS1, PTGS1*, PTGS2*, SCN5A*
Licorice	Isoglycyrol	AR, DPP4*, ESR1*, GSK3B, NOS2*, PIM1, PTGS2*
Licorice	Isolicoflavonol	AR, CALM1, CCNA2, CDK2, ESR1*, F10, F2*, GSK3B, HSP90AA1, NCOA2, NOS2*, PIM1, PPARG*, PRSS1, PTGS2*
Licorice	isorhamnetin	ACHE*, AKR1B1, AR, CALM1, camC, CCNA2, CDK2, CHEK1, DPP4*, ESR1*, ESR2, F2*, F7, GABRA1, GRIA2, GSK3B, HSP90AA1, MAOB*, MAPK14, NCF1, NCOA1, NCOA2, NOS2*, NOS3*, OLR1, PIK3CG*, PIM1, PPARD, PPARG*, PRKACA, PRSS1, PTGS1*, PTGS2*, PTPN1, PYGM, RELA*, XDH
Licorice	Isotrifoliol	AR, CCNA2, CDK2, CHEK1, ESR1*, ESR2, GSK3B, HSP90AA1, MAPK14, NOS2*, PIK3CG*, PIM1, PRKACA, PTGS2*
Licorice	Jaranol	AR, CALM1, CDK2, CHEK1, DPP4*, ESR2, HSP90AA1, NCOA2, NOS2*, PRSS1, PTGS1*, PTGS2*, SCN5A*
Licorice	Kanzonol F	AR, CALM1, ESR1*, ESR2, F10, NCOA2, PIM1, PTGS2*
Licorice	kanzonols W	AR, CALM1, CCNA2, CDK2, CHEK1, ESR1*, ESR2, F10, GSK3B, MAPK14, NCOA1, NCOA2, NOS2*, PIM1, PPARG*, PRSS1, PTGS1*, PTGS2*, RXRA, SCN5A*, TOP2A
Licorice	Licoagrocarpin	ACHE*, ADRA1B*, ADRB2*, AR, CALM1, CCNA2, CDK2, CHRM1, CHRM3*, CHRM5, ESR1*, ESR2, F10, F2*, GSK3B, HSP90AA1, KCNH2*, MAPK14, NCOA2, NOS2*, NOS3*, PIM1, PPARG*, PRSS1, PTGS1*, PTGS2*, RXRA, SCN5A*
Licorice	Licoagroisoflavone	AR, CALM1, CCNA2, CDK2, CHEK1, DPP4*, ESR1*, ESR2, F10, F2*, GSK3B, MAPK14, NOS2*, PIM1, PPARG*, PRSS1, PTGS2*, SCN5A*
Licorice	licochalcone a	ADRA1B*, ADRB2*, AR, BCL2*, CA2*, CALM1, CCNA2, CCND1, CDK2,

Herbal medicines	Chemical compounds	Targets
		CDK4, CHEK1, CHRM1, EIF6, ESR1*, ESR2, F10, FOSL2, GSK3B, HSP90AA1, MAPK1*, MAPK14, NCOA2, NOS2*, PIM1, PPARG*, PTGS1*, PTGS2*, RB1, RELA*, SCN5A*, SLC6A3, STAT3*
Licorice	Licochalcone B	ADRB2*, AR, CA2*, CALM1, CCNA2, CDK2, CHEK1, ESR1*, ESR2, GSK3B, HSP90AA1, MAPK14, NOS2*, PDE3A, PIM1, PPARG*, PRKACA, PTGS1*, PTGS2*
Licorice	licochalcone G	AR, CALM1, CCNA2, CDK2, ESR1*, ESR2, F10, GSK3B, HSP90AA1, IGHG1, KDR, MAPK14, NCOA2, NOS2*, PIM1, PPARG*, PTGS2*
Licorice	Licocoumarone	AR, CCNA2, CDK2, ESR1*, ESR2, GSK3B, HSP90AA1
Licorice	licoisoflavanone	ACHE*, AR, CALM1, CCNA2, CDK2, ESR1*, ESR2, F10, F7, GSK3B, HSP90AA1, NCOA1, NOS2*, PIM1, PPARG*, PRSS1, PTGS1*, PTGS2*, SCN5A*, TOP2A
Licorice	Licoisoflavone	AR, CALM1, CCNA2, CDK2, CHEK1, DPP4*, ESR1*, F10, F2*, HSP90AA1, KDR, MAPK14, NCOA2, NOS2*, PIM1, PPARG*, PRSS1, PTGS2*, TOP2A
Licorice	Licoisoflavone B	ACHE*, AR, CALM1, CCNA2, CDK2, CHEK1, ESR1*, ESR2, F10, F2*, GSK3B, NOS2*, PIM1, PPARG*, PRSS1, PTGS2*, TOP2A
Licorice	licopyranocoumarin	ACHE*, AR, CALM1, CCNA2, CDK2, ESR1*, F10, F2*, F7, KDR, NOS2*, PIM1, PPARG*, PRSS1, PTGS2*, TOP2A
Licorice	Licoricone	AR, CALM1, CHEK1, ESR1*, F10, F2*, KCNH2*, KDR, NCOA2, NOS2*, PIM1, PPARG*, PRSS1, PTGS2*, TOP2A
Licorice	Lupiwighteone	AR, CALM1, CCNA2, CDK2, CHEK1, DPP4*, ESR1*, ESR2, F10, F2*, GSK3B, HSP90AA1, MAPK14, NCOA2, NOS2*, PIM1, PPARG*, PRSS1, PTGS2*, SCN5A*, TOP2A
Licorice	Medicarpin	ADRA1A*, ADRA1B*, ADRA1D*, ADRB2*, CALM1, CCNA2, CDK2, CHRM1, CHRM2, CHRM3*, CHRM4, CHRM5, CHRNA7, DPP4*, DRD1, ESR1*, ESR2, HSP90AA1, HTR2A*, MAPK10, NOS2*, OPRD1*, OPRM1*, PDE3A, PIK3CG*,

Herbal medicines	Chemical compounds	Targets
		PIM1, PRKACA, PRSS1, PTGS1*, PTGS2*, RXRA, SCN5A*, SLC6A3, SLC6A4*
Licorice	naringenin	ABAT, ABCC1*, ADIPOQ, AKR1C1, AKT1*, ampC, APOB, BAD, BCL2*, CASP3*, CES1, CYP19A1, ESR1*, FASN, GOT1, GSR, HSP90AA1, LDLR, MAPK1*, MAPK3*, MTTP, mvaA, PIK3CG*, PLB1, PPARA*, PPARG*, PRKACA, PTGS1*, PTGS2*, RELA*, SOAT1, SOAT2, SOD1*, SREBF1, UGT1A1*
Licorice	Odoratin	AR, CALM1, CCNA2, CDK2, CHEK1, DPP4*, ESR1*, ESR2, GSK3B, HSP90AA1, MAPK14, NCOA2, NOS2*, PIM1, PPARG*, PRSS1, PTGS1*, PTGS2*, RXRA, SCN5A*
Licorice	Phaseol	AR, CCNA2, CDK2, CHEK1, ESR1*, F2*, GSK3B, HSP90AA1, KDR, MAPK14, PIM1, PPARG*, PRKACA, PTGS2*
Licorice	Phaseolinisoflavan	ACHE*, ADRA1B*, ADRB2*, AR, CALM1, CCNA2, CDK2, CHEK1, CHRM1, ESR1*, ESR2, F10, GSK3B, MAPK14, NCOA1, NOS2*, PIM1, PPARG*, PRSS1, PTGS2*, RXRA, SCN5A*
Licorice	quercetin	ABCG2*, ACACA, ACHE*, ACPP, ADRB2*, AHR, AHSA1, AKR1B1, AKT1*, ALOX5, AR, BAX*, BCL2*, BCL2L1*, BIRC5, CASP3*, CASP8, CASP9, CAV1, CCL2*, CCNB1, CCND1, CD40LG*, CDK1, CDKN1A, CDKN2A*, CHEK2, CHUK*, CLDN4*, COL3A1, CRP*, CTSD, CXCL10*, CXCL11, CXCL2, CXCL8*, CYP1A1*, CYP1B1, CYP3A4*, DCAF5, DIO1, DPP4*, DUOX2*, E2F1, E2F2, EGFR*, EIF6, ELK1, ERBB2, ERBB3, F10, F2*, F3*, F7, FOS*, GABRA1, GJA1*, GSTM1*, GSTM2, gyrB, HAS2, HERC5, HIF1A*, HK2, HSF1, HSP90AA1, HSPA5, HSPB1*, ICAM1, IFNG*, IGF2*, IGFBP3, IL10*, IL1A, IL1B*, IL2*, IL6*, INSR, IRF1, JUN*, KCNH2*, MAOB*, MAPK1*, MGAM*, MMP1, MMP2*, MMP3*, MMP9*, MPO*, MYC*, NCF1, NCOA2, NFE2L2, NFKBIA, NKX3-1, NOS3*, NOS3*, NPEPPS, NQO1, NR1I2*, NR1I3*, ODC1, PARP1, pbsA1, PCOLCE, PIK3CG*, PLAT, PLAU, PON1*, PPARA*, PPARD, PPARG*,

Herbal medicines	Chemical compounds	Targets
		PRKACA, PRKCA, PRKCB*, PRSS1, PRXC1A, PSMD3, PTEN, PTGER3, PTGS1*, PTGS2*, RAF1, RASA1, RASSF1, RB1, RELA*, RUNX1T1, RUNX2, RXRA, SCN5A*, SELE, SERPINE1*, SLC2A4, SOD1*, SPP1, STAT1, TGFB1*, THBD*, TNF*, TOP2A, topA, TP53*, VCAM1, XDH
Licorice	Quercetin der.	AR, CALM1, CDK2, DPP4*, ESR1*, ESR2, GSK3B, HSP90AA1, MAPK14, NCOA2, NOS2*, PPARG*, PRSS1, PTGS1*, PTGS2*, PTPN1, SCN5A*
Licorice	Semilicoisoflavone B	ACHE*, AR, CALM1, CDK2, CHEK1, ESR1*, F10, F2*, F7, GSK3B, HSP90AA1, NOS2*, PPARG*, PRSS1, PTGS2*, SCN5A*, TOP2A
Licorice	shinpterocarpin	ADRA1B*, ADRA1D*, ADRB2*, AR, CALM1, CCNA2, CDK2, CHRM1, CHRM3*, CHRNA7, ESR1*, ESR2, GSK3B, HTR3A*, KCNH2*, MAPK14, NCOA1, NOS2*, OPRD1*, OPRM1*, PIK3CG*, PIM1, PPARG*, PRKACA, PRSS1, PTGS1*, PTGS2*, RXRA, SCN5A*
Licorice	Sigmoidin-B	CALM1, ESR1*, F10, HSP90AA1, KDR, PTGS2*
Licorice	Vestitol	ADRA1A*, ADRA1B*, ADRB2*, AR, CALM1, CCNA2, CDK2, CHEK1, CHRM1, CHRM4, DPP4*, ESR1*, ESR2, GSK3B, HSP90AA1, HTR2A*, MAPK14, NOS2*, PDE3A, PIM1, PKIA, PPARG*, PRKACA, PRSS1, PTGS1*, PTGS2*, RXRA, SCN5A*, SLC6A3, SLC6A4*
Licorice	Xambioona	CALM1, ESR1*, ESR2, F10, NCOA2, NOS2*, PIM1, PTGS2*

*, Functional dyspepsia-associated targets.

Supplementary Table S4. Functional enrichment analyses for the functional dyspepsia-related targets of Jakyakgamcho-Tang.

	Category	Term	Count	Percent(%)	p-value
Gene ontology	Biological process	response to oxygen-containing compound	57	3.26	6.97E-35
		cellular response to chemical stimulus	69	2.00	1.55E-32
		response to chemical	75	1.55	3.05E-30
		cellular response to oxygen-containing compound	47	3.80	1.26E-29
		response to organic substance	66	1.91	1.06E-28
		inflammatory response	42	4.37	5.31E-28
		regulation of biological quality	69	1.68	1.94E-27
		response to lipid	40	4.18	1.32E-25
		response to external stimulus	60	1.93	1.47E-24
		response to lipopolysaccharide	28	8.26	1.82E-24
		response to molecule of bacterial origin	28	7.73	1.15E-23
		cellular response to organic substance	57	2.02	1.17E-23
		positive regulation of multicellular organismal process	45	3.01	1.26E-23
		response to nitrogen compound	41	3.48	2.70E-23
		regulation of multicellular organismal process	57	1.97	4.01E-23
		response to stress	66	1.53	1.02E-22
		regulation of cell population proliferation	47	2.65	1.17E-22
		response to organic cyclic compound	38	3.78	2.05E-22
		response to organonitrogen compound	39	3.57	3.06E-22
		cell population proliferation	49	2.38	6.30E-22
		defense response	48	2.45	7.43E-22
		positive regulation of biological process	75	1.16	3.89E-21
		response to stimulus	84	0.89	4.65E-21

	Category	Term	Count	Percent(%)	p-value
**Gene ontology**	**Biological process**	response to endogenous stimulus	45	2.60	5.30E-21
		response to drug	27	6.65	6.45E-21
		cytokine-mediated signaling pathway	34	4.13	1.14E-20
		biological process involved in interspecies interaction between organisms	45	2.55	1.34E-20
		regulation of localization	54	1.86	6.15E-20
		regulation of transport	45	2.45	6.82E-20
		homeostatic process	46	2.32	1.59E-19
		response to other organism	42	2.63	3.09E-19
		response to external biotic stimulus	42	2.62	3.17E-19
		positive regulation of response to stimulus	49	2.07	3.46E-19
		response to abiotic stimulus	38	3.01	7.57E-19
		response to biotic stimulus	42	2.56	7.82E-19
		positive regulation of molecular function	43	2.42	1.67E-18
		cellular response to cytokine stimulus	36	3.14	3.65E-18
		regulation of response to external stimulus	37	2.99	4.14E-18
		response to cytokine	37	2.98	4.62E-18
		regulation of molecular function	54	1.69	5.78E-18
		regulation of response to stress	41	2.52	5.86E-18
		positive regulation of cellular process	69	1.18	1.30E-17
		positive regulation of metabolic process	58	1.50	1.80E-17
		response to bacterium	30	4.04	2.06E-17
		cellular response to stimulus	77	0.98	2.24E-17
		cellular response to chemical stress	23	6.46	5.27E-17
		regulation of cell death	41	2.36	7.30E-17
		cellular response to nitrogen compound	29	3.98	1.57E-16

	Category	Term	Count	Percent(%)	p-value
**Gene ontology**	**Biological process**	positive regulation of gene expression	35	2.90	2.01E-16
		positive regulation of cell population proliferation	32	3.25	4.82E-16
		cell death	45	1.95	6.67E-16
		multicellular organismal process	76	0.95	8.82E-16
		positive regulation of macromolecule metabolic process	54	1.51	1.19E-15
		positive regulation of nitrogen compound metabolic process	51	1.62	1.46E-15
		regulation of response to stimulus	59	1.34	1.99E-15
		regulation of programmed cell death	38	2.37	2.87E-15
		cellular response to organonitrogen compound	27	4.02	3.13E-15
		response to wounding	27	3.97	4.39E-15
		regulation of immune system process	39	2.25	5.01E-15
		signaling	71	1.02	6.20E-15
		circulatory system process	26	4.11	9.13E-15
		regulation of apoptotic process	37	2.37	1.00E-14
		cellular response to endogenous stimulus	36	2.45	1.16E-14
		response to oxidative stress	23	5.03	1.39E-14
		positive regulation of response to external stimulus	24	4.63	1.55E-14
		response to inorganic substance	25	4.29	1.74E-14
		positive regulation of cellular metabolic process	51	1.53	2.23E-14
		response to reactive oxygen species	18	8.04	2.39E-14
		cell surface receptor signaling pathway	50	1.55	3.30E-14
		system process	43	1.86	3.97E-14
		cellular response to reactive oxygen species	16	10.06	4.96E-14
		cellular homeostasis	30	3.04	5.57E-14
		blood circulation	24	4.38	5.59E-14

	Category	Term	Count	Percent(%)	p-value
**Gene ontology**	**Biological process**	negative regulation of biological process	65	1.11	5.90E-14
		cell communication	70	1.01	6.41E-14
		regulation of multicellular organismal development	35	2.40	8.40E-14
		cellular response to organic cyclic compound	25	4.01	8.61E-14
		regulation of developmental process	45	1.72	1.04E-13
		negative regulation of apoptotic process	29	3.13	1.09E-13
		positive regulation of biosynthetic process	40	1.99	1.16E-13
		blood vessel diameter maintenance	15	11.03	1.33E-13
		regulation of tube diameter	15	11.03	1.33E-13
		regulation of tube size	15	10.95	1.48E-13
		negative regulation of programmed cell death	29	3.05	2.16E-13
		response to toxic substance	18	7.09	2.25E-13
		response to hormone	29	3.03	2.55E-13
		negative regulation of cell death	30	2.87	2.87E-13
		positive regulation of cell motility	24	4.05	3.21E-13
		cellular response to oxidative stress	19	6.21	3.35E-13
		acute inflammatory response	14	12.17	3.79E-13
		chemical homeostasis	32	2.58	3.82E-13
		ion homeostasis	27	3.32	4.03E-13
		positive regulation of small molecule metabolic process	15	10.20	4.36E-13
		positive regulation of cellular component movement	24	3.97	5.23E-13
		positive regulation of locomotion	24	3.95	5.64E-13
		response to extracellular stimulus	23	4.24	6.04E-13
		positive regulation of developmental process	33	2.44	6.08E-13
		apoptotic process	39	1.95	6.57E-13

	Category	Term	Count	Percent(%)	p-value
Gene ontology	Biological process	cation homeostasis	26	3.45	6.63E-13
		negative regulation of cellular process	60	1.16	8.01E-13
		inorganic ion homeostasis	26	3.39	9.68E-13
		positive regulation of catalytic activity	33	2.39	1.13E-12
		programmed cell death	40	1.85	1.31E-12
		positive regulation of cell migration	23	4.05	1.60E-12
		response to nutrient levels	22	4.29	2.34E-12
		positive regulation of signal transduction	35	2.15	2.41E-12
		regulation of DNA-binding transcription factor activity	21	4.60	2.97E-12
		positive regulation of cellular biosynthetic process	38	1.92	3.20E-12
		tube development	30	2.59	4.38E-12
		cellular response to cadmium ion	10	25.00	4.53E-12
		positive regulation of immune system process	29	2.69	5.78E-12
		vascular process in circulatory system	17	6.59	5.92E-12
		negative regulation of multicellular organismal process	30	2.56	6.33E-12
		cellular cation homeostasis	24	3.54	6.67E-12
		regulation of cell differentiation	35	2.07	7.59E-12
		positive regulation of phosphate metabolic process	28	2.79	7.80E-12
		positive regulation of phosphorus metabolic process	28	2.79	7.80E-12
		positive regulation of cell communication	36	1.99	9.10E-12
		cellular response to lipid	23	3.73	9.45E-12
		positive regulation of signaling	36	1.98	9.91E-12
		cellular ion homeostasis	24	3.46	1.08E-11
		wound healing	22	3.96	1.19E-11
		multi-multicellular organism process	16	7.05	1.47E-11

	Category	Term	Count	Percent(%)	p-value
**Gene ontology**	**Biological process**	circulatory system development	30	2.47	1.58E-11
		regulation of catalytic activity	41	1.67	1.68E-11
		regulation of inflammatory response	22	3.89	1.71E-11
		regulation of phosphorylation	32	2.26	1.84E-11
		positive regulation of transport	27	2.84	1.85E-11
		immune system process	48	1.40	1.88E-11
		regulation of phosphate metabolic process	34	2.07	2.27E-11
		regulation of phosphorus metabolic process	34	2.07	2.27E-11
		reactive oxygen species metabolic process	16	6.84	2.38E-11
		regulation of cellular component movement	29	2.52	2.95E-11
		regulation of ion transport	24	3.30	3.21E-11
		regulation of defense response	26	2.92	3.32E-11
		positive regulation of intracellular signal transduction	28	2.63	3.36E-11
		catabolic process	43	1.55	3.87E-11
		regulation of cell motility	28	2.62	3.96E-11
		cellular response to stress	38	1.77	4.45E-11
		transport	58	1.11	5.08E-11
		positive regulation of protein metabolic process	33	2.09	5.09E-11
		cellular response to lipopolysaccharide	15	7.43	5.17E-11
		metal ion homeostasis	23	3.43	5.63E-11
		anatomical structure morphogenesis	43	1.53	6.24E-11
		animal organ development	49	1.32	6.49E-11
		signal transduction	64	0.99	7.25E-11
		regulation of cell migration	27	2.68	7.60E-11
		positive regulation of nucleobase-containing compound metabolic process	36	1.85	8.33E-11

	Category	Term	Count	Percent(%)	p-value
**Gene ontology**	**Biological process**	positive regulation of vasoconstriction	9	25.71	9.24E-11
		cell activation	32	2.12	9.91E-11
		regulation of locomotion	28	2.52	1.06E-10
		regulation of transmembrane transport	22	3.55	1.14E-10
		regulation of system process	22	3.54	1.18E-10
		cell-cell signaling	34	1.96	1.19E-10
		cellular response to molecule of bacterial origin	15	6.94	1.39E-10
		response to mechanical stimulus	15	6.91	1.49E-10
		intracellular signal transduction	43	1.49	1.51E-10
		regulation of cell communication	48	1.32	1.71E-10
		phosphorylation	36	1.80	1.90E-10
		establishment of localization	58	1.08	1.98E-10
		positive regulation of cytokine production	20	4.02	1.98E-10
		regulation of intracellular signal transduction	35	1.86	2.05E-10
		cell migration	33	1.99	2.20E-10
		regulation of signaling	48	1.31	2.50E-10
		vasoconstriction	11	13.41	2.63E-10
		protein phosphorylation	33	1.97	2.74E-10
		negative regulation of developmental process	26	2.64	3.55E-10
		aging	17	5.11	3.93E-10
		developmental process	64	0.96	4.73E-10
		negative regulation of response to stimulus	35	1.81	4.83E-10
		system development	56	1.10	4.97E-10
		cell differentiation	52	1.18	5.14E-10
		regulation of blood pressure	14	7.29	5.25E-10

	Category	Term	Count	Percent(%)	p-value
Gene ontology	Biological process	regulation of leukocyte cell-cell adhesion	17	4.99	5.78E-10
		cellular metal ion homeostasis	21	3.51	5.82E-10
		ion transport	32	1.99	6.15E-10
		response to radiation	19	4.10	6.19E-10
		cell motility	34	1.85	6.53E-10
		localization of cell	34	1.85	6.53E-10
		localization	65	0.94	6.58E-10
		cellular response to biotic stimulus	15	6.22	6.93E-10
		multicellular organismal homeostasis	20	3.75	7.00E-10
		positive regulation of phosphorylation	25	2.69	7.66E-10
		female pregnancy	14	7.07	8.02E-10
		anatomical structure formation involved in morphogenesis	28	2.32	8.11E-10
		response to growth factor	23	2.99	9.79E-10
		cellular chemical homeostasis	24	2.81	1.03E-09
		cellular developmental process	52	1.16	1.06E-09
		muscle cell proliferation	15	6.00	1.18E-09
		response to peptide	20	3.63	1.29E-09
		response to cadmium ion	10	14.71	1.40E-09
		tube morphogenesis	25	2.62	1.48E-09
		regulation of protein phosphorylation	28	2.25	1.69E-09
		positive regulation of purine nucleotide metabolic process	9	19.15	1.71E-09
		positive regulation of nucleotide metabolic process	9	19.15	1.71E-09
		immune response	39	1.53	1.96E-09
		blood vessel morphogenesis	22	3.08	2.00E-09
		blood vessel development	23	2.89	2.05E-09

	Category	Term	Count	Percent(%)	p-value
Gene ontology	Biological process	regulation of protein modification process	32	1.90	2.13E-09
		negative regulation of cell population proliferation	23	2.86	2.46E-09
		gliogenesis	16	5.03	2.74E-09
		leukocyte cell-cell adhesion	17	4.51	2.91E-09
		response to metal ion	17	4.51	2.91E-09
		regulation of catabolic process	26	2.41	3.08E-09
		regulation of pri-miRNA transcription by RNA polymerase II	9	18.00	3.10E-09
		acute-phase response	9	18.00	3.10E-09
		movement of cell or subcellular component	37	1.60	3.20E-09
		gland development	18	4.06	3.38E-09
		positive regulation of smooth muscle cell proliferation	11	10.68	3.46E-09
		pri-miRNA transcription by RNA polymerase II	9	17.65	3.75E-09
		negative regulation of cell communication	30	2.00	3.84E-09
		regulation of smooth muscle cell proliferation	13	7.30	3.95E-09
		positive regulation of DNA-binding transcription factor activity	15	5.51	4.01E-09
		negative regulation of signaling	30	2.00	4.04E-09
		cellular response to growth factor stimulus	22	2.97	4.07E-09
		positive regulation of transcription by RNA polymerase II	27	2.25	4.81E-09
		positive regulation of transcription, DNA-templated	31	1.91	4.82E-09
		positive regulation of nucleic acid-templated transcription	31	1.91	4.82E-09
		smooth muscle cell proliferation	13	7.18	4.89E-09
		cellular response to inorganic substance	14	6.19	4.90E-09
		positive regulation of RNA biosynthetic process	31	1.91	4.90E-09
		regulation of protein metabolic process	40	1.45	5.13E-09
		vasculature development	23	2.76	5.22E-09

	Category	Term	Count	Percent(%)	p-value
**Gene ontology**	**Biological process**	positive regulation of macromolecule biosynthetic process	33	1.78	5.25E-09
		epithelial cell proliferation	18	3.91	6.36E-09
		positive regulation of protein phosphorylation	23	2.72	7.00E-09
		cellular catabolic process	37	1.55	7.17E-09
		regulation of cytokine production	23	2.72	7.17E-09
		apoptotic signaling pathway	20	3.28	8.10E-09
		cytokine production	23	2.69	8.48E-09
		regulation of transferase activity	25	2.41	8.71E-09
		positive regulation of cell death	21	3.04	9.25E-09
		regulation of signal transduction	43	1.32	9.98E-09
		angiogenesis	20	3.22	1.16E-08
		locomotion	34	1.67	1.22E-08
		multicellular organism development	57	1.01	1.31E-08
		MAPK cascade	24	2.49	1.39E-08
		cellular response to metal ion	13	6.57	1.53E-08
		regulation of cellular protein metabolic process	38	1.47	1.84E-08
		positive regulation of cellular protein metabolic process	29	1.95	1.99E-08
		positive regulation of cell differentiation	23	2.58	2.06E-08
		hematopoietic or lymphoid organ development	24	2.44	2.10E-08
		leukocyte differentiation	19	3.36	2.10E-08
		positive regulation of defense response	16	4.40	2.12E-08
		T cell activation	18	3.64	2.15E-08
		positive regulation of pri-miRNA transcription by RNA polymerase II	8	20.00	2.35E-08
		regulation of small molecule metabolic process	17	3.95	2.36E-08
		tissue development	34	1.62	2.55E-08

	Category	Term	Count	Percent(%)	p-value
Gene ontology	Biological process	regulation of vasoconstriction	9	14.06	3.24E-08
		export from cell	28	1.98	3.48E-08
		leukocyte migration	18	3.52	3.65E-08
		regulation of cell-cell adhesion	17	3.78	4.84E-08
		response to steroid hormone	16	4.16	4.92E-08
		positive regulation of RNA metabolic process	31	1.75	4.95E-08
		positive regulation of cytosolic calcium ion concentration	15	4.62	5.10E-08
		leukocyte proliferation	15	4.59	5.56E-08
		immune system development	24	2.31	6.28E-08
		phosphate-containing compound metabolic process	40	1.34	6.65E-08
		maintenance of location	15	4.45	8.51E-08
		phosphorus metabolic process	40	1.33	8.85E-08
		cellular response to abiotic stimulus	15	4.41	9.64E-08
		cellular response to environmental stimulus	15	4.41	9.64E-08
		regulation of T cell activation	15	4.40	1.00E-07
		negative regulation of cell differentiation	20	2.85	1.02E-07
		interleukin-8 production	10	9.62	1.10E-07
		regulation of interleukin-8 production	10	9.62	1.10E-07
		positive regulation of cell-cell adhesion	14	4.90	1.16E-07
		glial cell differentiation	13	5.58	1.19E-07
		positive regulation of inflammatory response	11	7.75	1.20E-07
		positive regulation of programmed cell death	19	3.03	1.26E-07
		secretion	28	1.88	1.26E-07
		anatomical structure development	58	0.94	1.27E-07
		response to estradiol	11	7.69	1.29E-07

	Category	Term	Count	Percent(%)	p-value
**Gene ontology**	**Biological process**	negative regulation of apoptotic signaling pathway	13	5.49	1.47E-07
		positive regulation of protein modification process	24	2.21	1.57E-07
		regulation of acute inflammatory response	8	16.00	1.58E-07
		ossification	16	3.83	1.67E-07
		regulation of blood circulation	14	4.76	1.67E-07
		reproductive process	28	1.85	1.72E-07
		reproduction	28	1.85	1.80E-07
		neuroinflammatory response	8	15.69	1.86E-07
		mononuclear cell migration	12	6.22	1.99E-07
		positive regulation of leukocyte cell-cell adhesion	13	5.35	2.01E-07
		response to glucocorticoid	11	7.38	2.02E-07
		regulation of cytosolic calcium ion concentration	15	4.18	2.07E-07
		response to UV	11	7.28	2.33E-07
		regulation of cellular catabolic process	22	2.41	2.38E-07
		regulation of protein serine/threonine kinase activity	17	3.40	2.50E-07
		regulation of kinase activity	22	2.39	2.75E-07
		biological regulation	82	0.64	2.83E-07
		organonitrogen compound metabolic process	61	0.88	2.95E-07
		synaptic signaling	20	2.68	3.16E-07
		regulation of leukocyte proliferation	13	5.14	3.32E-07
		heat generation	6	33.33	3.34E-07
		cell chemotaxis	14	4.52	3.37E-07
		nitric oxide biosynthetic process	9	10.84	3.60E-07
		regulation of cellular metabolic process	58	0.92	3.63E-07
		behavior	18	3.06	3.72E-07

	Category	Term	Count	Percent(%)	p-value
**Gene ontology**	**Biological process**	response to alcohol	13	5.08	3.84E-07
		regulation of cell adhesion	20	2.65	3.91E-07
		divalent inorganic cation homeostasis	17	3.30	3.95E-07
		peptidyl-serine phosphorylation	14	4.46	3.99E-07
		hydrogen peroxide metabolic process	8	14.29	4.08E-07
		T cell proliferation	12	5.83	4.23E-07
		regulation of metabolic process	62	0.86	4.29E-07
		regulation of body fluid levels	17	3.28	4.46E-07
		positive regulation of protein serine/threonine kinase activity	14	4.42	4.52E-07
		hemopoiesis	22	2.33	4.68E-07
		regulation of growth	19	2.79	4.85E-07
		negative regulation of response to external stimulus	18	3.00	5.15E-07
		negative regulation of metabolic process	42	1.20	5.35E-07
		regulation of leukocyte migration	12	5.66	5.90E-07
		nitric oxide metabolic process	9	10.23	6.14E-07
		regulation of immune response	24	2.07	6.20E-07
		positive regulation of transferase activity	19	2.75	6.20E-07
		regulation of neuron death	14	4.31	6.27E-07
		biosynthetic process	58	0.91	6.46E-07
		positive regulation of kinase activity	18	2.95	6.54E-07
		cellular calcium ion homeostasis	16	3.49	6.61E-07
		positive regulation of apoptotic process	18	2.95	6.71E-07
		reactive nitrogen species metabolic process	9	10.11	6.80E-07
		response to corticosteroid	11	6.55	7.35E-07
		growth	22	2.26	7.97E-07

	Category	Term	Count	Percent(%)	p-value
**Gene ontology**	**Biological process**	positive regulation of MAPK cascade	17	3.14	8.68E-07
		regulation of biosynthetic process	47	1.07	8.82E-07
		calcium ion homeostasis	16	3.40	9.63E-07
		muscle system process	16	3.40	9.63E-07
		regulation of epithelial cell proliferation	15	3.74	9.64E-07
		peptidyl-serine modification	14	4.15	1.01E-06
		epithelial cell apoptotic process	10	7.69	1.02E-06
		regulation of systemic arterial blood pressure by norepinephrine-epinephrine	5	50.00	1.07E-06
		temperature homeostasis	11	6.32	1.07E-06
		chemical synaptic transmission	19	2.66	1.07E-06
		anterograde trans-synaptic signaling	19	2.66	1.07E-06
		positive regulation of interleukin-8 production	8	12.70	1.08E-06
		cell-cell adhesion	21	2.34	1.19E-06
		extrinsic apoptotic signaling pathway	12	5.31	1.23E-06
		regulation of protein kinase activity	20	2.47	1.28E-06
		leukocyte chemotaxis	12	5.29	1.29E-06
		peptidyl-amino acid modification	25	1.91	1.29E-06
		trans-synaptic signaling	19	2.64	1.30E-06
		response to temperature stimulus	12	5.24	1.43E-06
		anatomical structure homeostasis	16	3.29	1.52E-06
		regulation of MAPK cascade	19	2.59	1.74E-06
		positive regulation of purine nucleotide biosynthetic process	6	26.09	1.78E-06
		positive regulation of nucleotide biosynthetic process	6	26.09	1.78E-06
		regulation of secretion	18	2.77	1.81E-06
		cellular divalent inorganic cation homeostasis	16	3.23	2.04E-06

	Category	Term	Count	Percent(%)	p-value
**Gene ontology**	**Biological process**	regulation of hemopoiesis	15	3.54	2.08E-06
		leukocyte activation	25	1.86	2.14E-06
		positive regulation of ATP metabolic process	7	16.28	2.28E-06
		positive regulation of gliogenesis	8	11.59	2.29E-06
		regulation of cell activation	18	2.72	2.42E-06
		regulation of angiogenesis	14	3.88	2.46E-06
		secretion by cell	25	1.85	2.50E-06
		phospholipase C-activating G protein-coupled receptor signaling pathway	9	8.74	2.55E-06
		superoxide metabolic process	8	11.43	2.57E-06
		mononuclear cell proliferation	13	4.35	2.58E-06
		regulation of binding	14	3.85	2.74E-06
		reproductive structure development	15	3.46	2.77E-06
		positive regulation of ion transport	13	4.32	2.80E-06
		neuron death	14	3.84	2.84E-06
		regulation of apoptotic signaling pathway	14	3.84	2.84E-06
		response to virus	14	3.83	2.94E-06
		reproductive system development	15	3.44	3.04E-06
		regulation of vasculature development	14	3.81	3.05E-06
		regulation of secretion by cell	17	2.88	3.24E-06
		signal transduction in absence of ligand	8	11.11	3.24E-06
		extrinsic apoptotic signaling pathway in absence of ligand	8	11.11	3.24E-06
		negative regulation of cellular metabolic process	36	1.29	3.30E-06
		placenta development	10	6.80	3.40E-06
		mononuclear cell differentiation	15	3.41	3.45E-06
		calcium ion transport	15	3.40	3.56E-06

	Category	Term	Count	Percent(%)	p-value
**Gene ontology**	**Biological process**	rhythmic process	13	4.23	3.56E-06
		regulation of gliogenesis	9	8.41	3.59E-06
		regulation of ion transmembrane transport	16	3.08	4.03E-06
		regulation of membrane potential	15	3.36	4.15E-06
		metal ion transport	16	3.06	4.38E-06
		regulation of neuroinflammatory response	7	14.89	4.38E-06
		regulation of biological process	79	0.65	4.75E-06
		negative regulation of signal transduction	25	1.79	4.76E-06
		lymphocyte activation	19	2.44	4.77E-06
		response to purine-containing compound	10	6.54	5.03E-06
		animal organ morphogenesis	22	2.04	5.51E-06
		regulation of reactive oxygen species metabolic process	10	6.45	5.71E-06
		regulation of mononuclear cell migration	9	7.89	6.32E-06
		transmembrane transport	27	1.64	6.34E-06
		positive regulation of protein kinase activity	16	2.98	6.39E-06
		response to peptide hormone	15	3.25	6.50E-06
		regulation of cellular process	77	0.67	6.71E-06
		negative regulation of catabolic process	13	4.01	6.82E-06
		organic acid metabolic process	22	2.00	7.61E-06
		cellular response to mechanical stimulus	8	10.00	7.62E-06
		response to light stimulus	13	3.96	7.91E-06
		regulation of systemic arterial blood pressure mediated by a chemical signal	7	13.73	7.95E-06
		regulation of cation transmembrane transport	14	3.54	8.10E-06
		positive regulation of acute inflammatory response	6	20.69	8.20E-06
		response to UV-A	5	35.71	8.35E-06

	Category	Term	Count	Percent(%)	p-value
**Gene ontology**	**Biological process**	cation transport	23	1.90	8.37E-06
		platelet activation	10	6.13	9.30E-06
		astrocyte differentiation	8	9.64	1.02E-05
		regulation of metal ion transport	12	4.40	1.05E-05
		cellular response to toxic substance	9	7.44	1.07E-05
		regulation of purine nucleotide metabolic process	9	7.44	1.07E-05
		regulation of autophagy	13	3.86	1.10E-05
		cellular biosynthetic process	55	0.89	1.14E-05
		regulation of leukocyte chemotaxis	9	7.38	1.15E-05
		regulation of generation of precursor metabolites and energy	10	5.99	1.18E-05
		positive regulation of hydrolase activity	18	2.46	1.20E-05
		regulation of nucleotide metabolic process	9	7.32	1.24E-05
		response to oxygen levels	14	3.41	1.26E-05
		myeloid leukocyte migration	11	5.00	1.28E-05
		nervous system process	25	1.71	1.33E-05
		positive regulation of T cell activation	11	4.98	1.34E-05
		granulocyte chemotaxis	9	7.20	1.43E-05
		positive regulation of heart rate by epinephrine-norepinephrine	4	66.67	1.45E-05
		signal release	15	3.06	1.48E-05
		regulation of protein transport	16	2.81	1.49E-05
		regulation of response to wounding	10	5.81	1.56E-05
		regulation of hydrolase activity	23	1.84	1.60E-05
		monocarboxylic acid metabolic process	17	2.56	1.81E-05
		regulation of chemotaxis	11	4.82	1.85E-05
		cellular response to external stimulus	13	3.68	1.91E-05

	Category	Term	Count	Percent(%)	p-value
**Gene ontology**	**Biological process**	DNA-templated transcription, initiation	12	4.15	1.99E-05
		regulation of T cell proliferation	10	5.65	2.06E-05
		regulation of leukocyte differentiation	12	4.14	2.07E-05
		response to nutrient	10	5.62	2.17E-05
		multi-organism reproductive process	21	1.98	2.29E-05
		positive regulation of neurogenesis	11	4.72	2.32E-05
		ion transmembrane transport	22	1.88	2.39E-05
		regulation of mononuclear cell proliferation	11	4.70	2.42E-05
		oxoacid metabolic process	21	1.97	2.53E-05
		production of molecular mediator involved in inflammatory response	8	8.60	2.55E-05
		hydrogen peroxide biosynthetic process	5	29.41	2.55E-05
		modulation of chemical synaptic transmission	14	3.23	2.59E-05
		lymphocyte proliferation	12	4.05	2.60E-05
		adenylate cyclase-modulating G protein-coupled receptor signaling pathway	11	4.66	2.65E-05
		muscle contraction	13	3.58	2.66E-05
		regulation of trans-synaptic signaling	14	3.22	2.67E-05
		regulation of establishment of protein localization	16	2.69	2.73E-05
		myeloid cell differentiation	14	3.21	2.75E-05
		reactive oxygen species biosynthetic process	7	11.48	2.89E-05
		regulation of cellular biosynthetic process	44	1.02	2.94E-05
		response to organophosphorus	9	6.62	3.00E-05
		response to hypoxia	13	3.54	3.03E-05
		regulation of cellular component organization	32	1.31	3.09E-05
		regulation of anatomical structure size	15	2.89	3.19E-05
		positive regulation of cell adhesion	14	3.17	3.26E-05

	Category	Term	Count	Percent(%)	p-value
**Gene ontology**	**Biological process**	response to vitamin	8	8.33	3.28E-05
		positive regulation of fever generation	4	57.14	3.38E-05
		positive regulation of blood pressure by epinephrine-norepinephrine	4	57.14	3.38E-05
		positive regulation of leukocyte migration	9	6.52	3.41E-05
		regulation of wound healing	9	6.52	3.41E-05
		positive regulation of transmembrane transport	11	4.55	3.43E-05
		negative regulation of nitrogen compound metabolic process	33	1.27	3.45E-05
		tissue remodeling	10	5.35	3.48E-05
		cellular response to interleukin-1	10	5.35	3.48E-05
		negative regulation of gene silencing by miRNA	5	27.78	3.52E-05
		regulation of lymphocyte activation	15	2.87	3.54E-05
		regulation of endothelial cell proliferation	10	5.32	3.67E-05
		regulation of signaling receptor activity	10	5.32	3.67E-05
		response to fluid shear stress	6	16.22	3.90E-05
		small molecule metabolic process	28	1.46	4.03E-05
		positive regulation of vasculature development	10	5.26	4.05E-05
		positive regulation of angiogenesis	10	5.26	4.05E-05
		response to ethanol	9	6.38	4.12E-05
		cellular metabolic process	74	0.68	4.24E-05
		organic cyclic compound metabolic process	54	0.87	4.58E-05
		positive regulation of chemotaxis	9	6.29	4.66E-05
		negative regulation of posttranscriptional gene silencing	5	26.32	4.76E-05
		negative regulation of gene silencing by RNA	5	26.32	4.76E-05
		response to decreased oxygen levels	13	3.40	4.87E-05
		enzyme linked receptor protein signaling pathway	21	1.90	4.88E-05

	Category	Term	Count	Percent(%)	p-value
**Gene ontology**	**Biological process**	lipid metabolic process	24	1.66	4.97E-05
		regulation of systemic arterial blood pressure	8	7.84	5.31E-05
		nitrogen compound transport	30	1.35	5.34E-05
		negative regulation of molecular function	22	1.79	5.82E-05
		regulation of protein localization	19	2.08	6.03E-05
		regulation of DNA metabolic process	13	3.34	6.03E-05
		positive regulation of myeloid cell differentiation	8	7.69	6.19E-05
		sensory perception of pain	8	7.69	6.19E-05
		regulation of monooxygenase activity	7	10.29	6.25E-05
		adenylate cyclase-activating G protein-coupled receptor signaling pathway	9	6.08	6.29E-05
		regulation of nitrogen compound metabolic process	52	0.88	6.42E-05
		granulocyte migration	9	6.04	6.67E-05
		regulation of nitric oxide biosynthetic process	7	10.14	6.93E-05
		regulation of cellular response to stress	17	2.34	7.02E-05
		regulation of myeloid cell differentiation	11	4.23	7.18E-05
		response to ketone	10	4.93	7.61E-05
		regulation of epithelial cell apoptotic process	8	7.48	7.74E-05
		amide transport	12	3.67	7.82E-05
		endothelial cell proliferation	10	4.90	7.97E-05
		organic cyclic compound biosynthetic process	44	0.99	8.12E-05
		carboxylic acid metabolic process	20	1.94	8.15E-05
		negative regulation of extrinsic apoptotic signaling pathway	8	7.41	8.33E-05
		leukocyte apoptotic process	8	7.41	8.33E-05
		regulation of calcium ion transport	11	4.17	8.40E-05
		regulation of nitric oxide metabolic process	7	9.86	8.48E-05

	Category	Term	Count	Percent(%)	p-value
Gene ontology	Biological process	response to tumor necrosis factor	12	3.61	9.24E-05
		regulation of cytokine production involved in inflammatory response	7	9.72	9.35E-05
		cytokine production involved in inflammatory response	7	9.72	9.35E-05
		positive regulation of lipid metabolic process	9	5.81	9.39E-05
		regulation of purine nucleotide biosynthetic process	6	13.95	9.99E-05
		multi-organism process	21	1.82	1.01E-04
		cell adhesion	24	1.60	1.03E-04
		regulation of anatomical structure morphogenesis	20	1.91	1.03E-04
		cellular response to peptide	13	3.19	1.05E-04
		negative regulation of lipid storage	5	22.73	1.07E-04
		regulation of killing of cells of other organism	5	22.73	1.07E-04
		positive regulation of glial cell proliferation	5	22.73	1.07E-04
		negative regulation of transport	14	2.87	1.10E-04
		cellular aromatic compound metabolic process	52	0.87	1.11E-04
		organic substance catabolic process	30	1.31	1.12E-04
		biological adhesion	24	1.59	1.12E-04
		tissue homeostasis	11	4.04	1.14E-04
		regulation of nucleotide biosynthetic process	6	13.64	1.15E-04
		regulation of gene expression	48	0.92	1.16E-04
		cellular detoxification	8	7.08	1.19E-04
		regulation of cellular localization	18	2.12	1.19E-04
		positive regulation of epithelial cell proliferation	10	4.69	1.20E-04
		regulation of fever generation	4	44.44	1.21E-04
		stress-activated MAPK cascade	11	4.01	1.23E-04
		positive regulation of hemopoiesis	9	5.63	1.24E-04

	Category	Term	Count	Percent(%)	p-value
**Gene ontology**	**Biological process**	positive regulation of leukocyte differentiation	9	5.63	1.24E-04
		collagen metabolic process	8	7.02	1.27E-04
		negative regulation of blood pressure	6	13.33	1.32E-04
		positive regulation of nitric oxide biosynthetic process	6	13.33	1.32E-04
		ameboidal-type cell migration	14	2.82	1.38E-04
		response to interleukin-1	10	4.61	1.43E-04
		cellular localization	38	1.08	1.46E-04
		regulation of macromolecule metabolic process	55	0.83	1.48E-04
		circadian rhythm	10	4.59	1.49E-04
		positive regulation of nitric oxide metabolic process	6	13.04	1.52E-04
		positive regulation of NF-kappaB transcription factor activity	9	5.49	1.53E-04
		positive regulation of nervous system development	11	3.91	1.59E-04
		heart contraction	11	3.90	1.65E-04
		response to alkaloid	8	6.78	1.67E-04
		myeloid leukocyte differentiation	10	4.52	1.69E-04
		blood coagulation	12	3.42	1.70E-04
		regulation of production of miRNAs involved in gene silencing by miRNA	5	20.83	1.71E-04
		regulation of production of small RNA involved in gene silencing by RNA	5	20.83	1.71E-04
		cellular response to hormone stimulus	16	2.35	1.78E-04
		animal organ regeneration	7	8.86	1.79E-04
		regulation of cell development	14	2.75	1.89E-04
		establishment of localization in cell	33	1.19	1.91E-04
		hemostasis	12	3.38	1.92E-04
		regulation of primary metabolic process	52	0.86	2.02E-04
		peptide transport	11	3.82	2.04E-04

	Category	Term	Count	Percent(%)	p-value
Gene ontology	Biological process	regulation of peptide transport	10	4.42	2.09E-04
		coagulation	12	3.35	2.10E-04
		positive regulation of carbohydrate metabolic process	7	8.64	2.14E-04
		positive regulation of calcidiol 1-monooxygenase activity	3	100.00	2.18E-04
		stress-activated protein kinase signaling cascade	11	3.79	2.18E-04
		regulation of cellular respiration	6	12.24	2.24E-04
		positive regulation of vascular associated smooth muscle cell proliferation	6	12.24	2.24E-04
		regulation of myeloid leukocyte differentiation	8	6.50	2.30E-04
		heart process	11	3.77	2.34E-04
		regulation of protein secretion	11	3.75	2.42E-04
		organic substance transport	32	1.20	2.43E-04
		negative regulation of wound healing	7	8.43	2.53E-04
		defense response to other organism	21	1.72	2.65E-04
		epithelium development	22	1.64	2.66E-04
		regulation of lymphocyte proliferation	10	4.31	2.67E-04
		positive regulation of lymphocyte activation	12	3.28	2.67E-04
		intrinsic apoptotic signaling pathway	11	3.70	2.78E-04
		cognition	11	3.70	2.78E-04
		receptor signaling pathway via JAK-STAT	9	5.11	2.80E-04
		negative regulation of immune system process	15	2.44	2.90E-04
		icosanoid metabolic process	8	6.30	2.95E-04
		regulation of leukocyte activation	15	2.43	3.09E-04
		epithelial cell migration	12	3.23	3.10E-04
		positive regulation of catabolic process	14	2.64	3.11E-04
		positive regulation of heat generation	4	36.36	3.14E-04

	Category	Term	Count	Percent(%)	p-value
Gene ontology	Biological process	fever generation	4	36.36	3.14E-04
		metabolic process	76	0.64	3.17E-04
		regulation of nucleobase-containing compound metabolic process	41	0.99	3.22E-04
		regulation of neurogenesis	12	3.22	3.28E-04
		epithelium migration	12	3.21	3.38E-04
		regulation of nervous system development	13	2.87	3.55E-04
		regulation of hormone levels	14	2.60	3.73E-04
		positive regulation of cell development	11	3.59	3.75E-04
		regulation of epithelial cell migration	11	3.59	3.75E-04
		positive regulation of lipid biosynthetic process	7	7.95	3.80E-04
		tissue migration	12	3.16	4.01E-04
		regulation of nitric-oxide synthase activity	6	11.11	4.06E-04
		organ growth	9	4.89	4.10E-04
		positive regulation of cellular catabolic process	13	2.83	4.23E-04
		regulation of vesicle-mediated transport	14	2.57	4.27E-04
		leukocyte homeostasis	7	7.78	4.44E-04
		receptor signaling pathway via STAT	9	4.84	4.49E-04
		regulation of heart contraction	10	4.05	4.78E-04
		positive regulation of protein localization to nucleus	7	7.69	4.79E-04
		lymphocyte differentiation	12	3.10	4.88E-04
		embryo implantation	6	10.71	5.07E-04
		response to amyloid-beta	6	10.71	5.07E-04
		vascular associated smooth muscle cell proliferation	7	7.61	5.16E-04
		regulation of vascular associated smooth muscle cell proliferation	7	7.61	5.16E-04
		positive regulation of ion transmembrane transport	9	4.74	5.38E-04

	Category	Term	Count	Percent(%)	p-value
Gene ontology	Biological process	regulation of lipid metabolic process	12	3.07	5.45E-04
		negative regulation of protein binding	7	7.53	5.56E-04
		adenylate cyclase-activating adrenergic receptor signaling pathway	5	16.67	5.61E-04
		lipopolysaccharide-mediated signaling pathway	6	10.53	5.65E-04
		central nervous system development	19	1.81	5.66E-04
		positive regulation of leukocyte chemotaxis	7	7.37	6.44E-04
		chemotaxis	15	2.29	6.53E-04
		learning or memory	10	3.91	6.65E-04
		regulation of muscle system process	10	3.89	6.90E-04
		taxis	15	2.28	6.92E-04
		nervous system development	30	1.21	6.99E-04
		regulation of proteolysis	16	2.12	7.34E-04
		detoxification	8	5.59	7.38E-04
		positive regulation of immune response	16	2.11	7.47E-04
		positive regulation of myeloid leukocyte differentiation	6	10.00	7.71E-04
		positive regulation of wound healing	6	10.00	7.71E-04
		transmembrane receptor protein tyrosine kinase signaling pathway	16	2.11	7.74E-04
		negative regulation of leukocyte cell-cell adhesion	8	5.56	7.78E-04
		negative regulation of gene silencing	5	15.63	7.87E-04
		positive regulation of peptidyl-tyrosine phosphorylation	9	4.52	7.96E-04
		protein metabolic process	50	0.85	8.05E-04
		negative regulation of macromolecule metabolic process	35	1.07	8.10E-04
		biological process involved in symbiotic interaction	11	3.32	8.22E-04
		fatty acid metabolic process	12	2.95	8.36E-04
		negative regulation of response to wounding	7	7.07	8.55E-04

	Category	Term	Count	Percent(%)	p-value
Gene ontology	Biological process	regulation of peptidyl-serine phosphorylation	8	5.48	8.65E-04
		positive regulation of DNA metabolic process	9	4.48	8.66E-04
		heterocycle biosynthetic process	41	0.96	9.11E-04
		negative regulation of transmembrane transport	8	5.44	9.11E-04
		regulation of phagocytosis	7	7.00	9.16E-04
		DNA metabolic process	18	1.84	9.19E-04
		positive regulation of monooxygenase activity	5	15.15	9.24E-04
		maternal process involved in female pregnancy	6	9.68	9.39E-04
		positive regulation of ATP biosynthetic process	4	28.57	9.43E-04
		negative regulation of production of miRNAs involved in gene silencing by miRNA	4	28.57	9.43E-04
		regulation of heat generation	4	28.57	9.43E-04
		aromatic compound biosynthetic process	41	0.96	9.74E-04
		B cell proliferation	7	6.93	9.80E-04
		positive regulation of leukocyte activation	12	2.90	1.00E-03
		modulation of process of other organism	8	5.37	1.01E-03
		neurogenesis	24	1.41	1.02E-03
		lymphocyte homeostasis	6	9.52	1.03E-03
		liver development	8	5.33	1.06E-03
		generation of neurons	23	1.46	1.08E-03
		regulation of peptidyl-tyrosine phosphorylation	10	3.70	1.09E-03
		regulation of endothelial cell apoptotic process	6	9.38	1.14E-03
		negative regulation of protein metabolic process	20	1.64	1.14E-03
		homeostasis of number of cells	10	3.66	1.20E-03
		G protein-coupled receptor signaling pathway	21	1.57	1.21E-03
		hepaticobiliary system development	8	5.23	1.24E-03

	Category	Term	Count	Percent(%)	p-value
**Gene ontology**	**Biological process**	adrenergic receptor signaling pathway	5	14.29	1.25E-03
		maternal placenta development	5	14.29	1.25E-03
		regulation of heart rate	7	6.67	1.28E-03
		positive regulation of cell activation	12	2.82	1.36E-03
		positive regulation of leukocyte proliferation	8	5.16	1.37E-03
		regulation of transcription by RNA polymerase II	30	1.17	1.43E-03
		macromolecule modification	41	0.94	1.44E-03
		negative regulation of neuron death	9	4.21	1.47E-03
		regulation of carbohydrate metabolic process	9	4.19	1.52E-03
		viral process	18	1.78	1.54E-03
		negative regulation of cell activation	9	4.17	1.59E-03
		negative regulation of lipid localization	6	8.82	1.64E-03
		positive regulation of phagocytosis	6	8.82	1.64E-03
		regulation of cysteine-type endopeptidase activity involved in apoptotic process	9	4.15	1.65E-03
		smooth muscle contraction	7	6.42	1.65E-03
		regulation of cell killing	7	6.42	1.65E-03
		positive regulation of peptidyl-serine phosphorylation	7	6.42	1.65E-03
		regulation of extrinsic apoptotic signaling pathway	8	5.03	1.66E-03
		liver regeneration	5	13.51	1.67E-03
		negative regulation of binding	8	5.00	1.74E-03
		nucleobase-containing compound biosynthetic process	40	0.95	1.77E-03
		regulation of neuron apoptotic process	9	4.11	1.78E-03
		endothelial cell apoptotic process	6	8.70	1.78E-03
		phosphatidylinositol 3-kinase signaling	8	4.97	1.82E-03
		regulation of transcription, DNA-templated	36	1.02	1.83E-03

	Category	Term	Count	Percent(%)	p-value
Gene ontology	Biological process	regulation of peptide hormone secretion	9	4.09	1.85E-03
		regulation of nucleic acid-templated transcription	36	1.02	1.86E-03
		regulation of organ growth	7	6.31	1.87E-03
		superoxide anion generation	5	13.16	1.92E-03
		regulation of RNA biosynthetic process	36	1.02	1.93E-03
		mammary gland epithelium development	6	8.57	1.94E-03
		protein kinase B signaling	10	3.46	2.02E-03
		I-kappaB kinase/NF-kappaB signaling	10	3.45	2.08E-03
		negative regulation of synaptic transmission	6	8.45	2.12E-03
		regulation of peptide secretion	9	4.02	2.15E-03
		neuron-glial cell signaling	3	60.00	2.17E-03
		response to food	5	12.82	2.19E-03
		regulation of glial cell proliferation	5	12.82	2.19E-03
		T cell homeostasis	5	12.82	2.19E-03
		sequestering of triglyceride	4	23.53	2.22E-03
		organic substance biosynthetic process	51	0.81	2.32E-03
		transcription initiation from RNA polymerase II promoter	9	3.96	2.40E-03
		Fc-epsilon receptor signaling pathway	8	4.79	2.40E-03
		regulation of intrinsic apoptotic signaling pathway	8	4.79	2.40E-03
		postsynaptic signal transduction	5	12.50	2.50E-03
		regulation of immunoglobulin production	6	8.22	2.50E-03
		positive regulation of tyrosine phosphorylation of STAT protein	6	8.22	2.50E-03
		regulation of oxidoreductase activity	7	6.03	2.51E-03
		cation transmembrane transport	17	1.82	2.52E-03
		heterocycle metabolic process	49	0.83	2.69E-03

	Category	Term	Count	Percent(%)	p-value
Gene ontology	Biological process	positive regulation of response to wounding	6	8.11	2.71E-03
		chronic inflammatory response	4	22.22	2.84E-03
		regulation of muscle contraction	8	4.68	2.87E-03
		regulation of mitochondrial membrane potential	6	8.00	2.93E-03
		protein modification process	39	0.95	2.94E-03
		cellular protein modification process	39	0.95	2.94E-03
		positive regulation of cellular amide metabolic process	8	4.65	3.00E-03
		positive regulation of MAP kinase activity	9	3.85	3.09E-03
		regulation of ATP metabolic process	7	5.83	3.16E-03
		regulation of DNA binding	7	5.83	3.16E-03
		positive regulation of cation transmembrane transport	8	4.60	3.27E-03
		protein secretion	11	2.89	3.28E-03
		establishment of protein localization to extracellular region	11	2.88	3.36E-03
		killing of cells of other organism	6	7.79	3.43E-03
		positive regulation of proteolysis	11	2.87	3.45E-03
		regulation of macromolecule biosynthetic process	39	0.94	3.48E-03
		positive regulation of cellular protein localization	10	3.26	3.49E-03
		cellular nitrogen compound metabolic process	52	0.79	3.51E-03
		regulation of peptidase activity	12	2.57	3.56E-03
		cellular response to tumor necrosis factor	10	3.25	3.59E-03
		regulation of oxidative stress-induced cell death	6	7.69	3.70E-03
		glial cell development	7	5.69	3.73E-03
		negative regulation of intracellular signal transduction	13	2.33	3.74E-03
		peptidyl-tyrosine phosphorylation	11	2.84	3.82E-03
		developmental growth	14	2.14	3.86E-03

	Category	Term	Count	Percent(%)	p-value
**Gene ontology**	**Biological process**	multicellular organismal reproductive process	16	1.86	3.91E-03
		cellular lipid metabolic process	18	1.67	3.92E-03
		regulation of cysteine-type endopeptidase activity	9	3.73	3.94E-03
		protein localization to extracellular region	11	2.83	4.01E-03
		peptidyl-tyrosine modification	11	2.82	4.11E-03
		autophagy	13	2.30	4.21E-03
		process utilizing autophagic mechanism	13	2.30	4.21E-03
		apoptotic mitochondrial changes	7	5.56	4.39E-03
		positive regulation of epithelial cell migration	8	4.42	4.40E-03
		I-kappaB phosphorylation	4	20.00	4.47E-03
		positive regulation of amyloid-beta formation	4	20.00	4.47E-03
		cell development	26	1.23	4.54E-03
		response to nicotine	5	11.11	4.55E-03
		positive regulation of signaling receptor activity	5	11.11	4.55E-03
		regulation of lipid localization	8	4.40	4.58E-03
		regulation of glycolytic process	6	7.41	4.62E-03
		regulation of transporter activity	10	3.15	4.65E-03
		proteolysis	24	1.30	4.81E-03
		regulation of RNA metabolic process	37	0.96	4.96E-03
		drug metabolic process	5	10.87	5.09E-03
		positive regulation of receptor signaling pathway via JAK-STAT	5	10.87	5.09E-03
		cellular protein metabolic process	45	0.85	5.14E-03
		multicellular organism reproduction	16	1.82	5.27E-03
		transcription by RNA polymerase II	30	1.10	5.30E-03
		regulation of I-kappaB kinase/NF-kappaB signaling	9	3.60	5.33E-03

	Category	Term	Count	Percent(%)	p-value
**Gene ontology**	**Biological process**	cellular component disassembly	13	2.25	5.41E-03
		positive regulation of cyclase activity	4	19.05	5.50E-03
		negative regulation of glucose transmembrane transport	4	19.05	5.50E-03
		neuron apoptotic process	9	3.59	5.50E-03
		regulation of insulin secretion	8	4.28	5.61E-03
		negative regulation of growth	9	3.54	6.07E-03
		cytosolic calcium ion transport	8	4.23	6.08E-03
		transcription, DNA-templated	36	0.97	6.22E-03
		nucleic acid-templated transcription	36	0.97	6.30E-03
		response to cold	5	10.42	6.31E-03
		positive regulation of protein transport	10	3.04	6.48E-03
		regulation of lipid biosynthetic process	8	4.19	6.57E-03
		regulation of DNA-templated transcription, initiation	6	6.98	6.57E-03
		lipid storage	6	6.98	6.57E-03
		regulation of cell cycle	18	1.61	6.64E-03
		vitamin D metabolic process	4	18.18	6.70E-03
		regulation of ATP biosynthetic process	4	18.18	6.70E-03
		ERK1 and ERK2 cascade	10	3.02	6.84E-03
		RNA biosynthetic process	36	0.96	6.99E-03
		peptide hormone secretion	9	3.47	7.11E-03
		organic substance metabolic process	72	0.63	7.20E-03
		response to acid chemical	7	5.15	7.30E-03
		steroid metabolic process	10	2.99	7.41E-03
		activation of protein kinase activity	10	2.99	7.41E-03
		negative regulation of mononuclear cell proliferation	6	6.82	7.52E-03

	Category	Term	Count	Percent(%)	p-value
**Gene ontology**	**Biological process**	regulation of calcidiol 1-monooxygenase activity	3	42.86	7.54E-03
		regulation of chronic inflammatory response	3	42.86	7.54E-03
		response to epidermal growth factor	5	10.00	7.75E-03
		positive regulation of receptor signaling pathway via STAT	5	10.00	7.75E-03
		cell killing	8	4.08	7.96E-03
		regulation of protein binding	8	4.08	7.96E-03
		immune effector process	18	1.59	8.01E-03
		regulation of tyrosine phosphorylation of STAT protein	6	6.74	8.04E-03
		positive regulation of hemostasis	4	17.39	8.08E-03
		regulation of superoxide anion generation	4	17.39	8.08E-03
		positive regulation of blood coagulation	4	17.39	8.08E-03
		regulation of developmental growth	10	2.96	8.23E-03
		phosphatidylinositol-mediated signaling	8	4.06	8.27E-03
		cellular response to radiation	8	4.06	8.27E-03
		T cell differentiation	9	3.41	8.32E-03
		vesicle-mediated transport	26	1.19	8.42E-03
		digestive system development	7	5.04	8.44E-03
		regulation of cellular macromolecule biosynthetic process	38	0.93	8.44E-03
		cellular response to peptide hormone stimulus	10	2.95	8.45E-03
		regulation of granulocyte chemotaxis	5	9.80	8.56E-03
		negative regulation of reactive oxygen species metabolic process	5	9.80	8.56E-03
		production of miRNAs involved in gene silencing by miRNA	5	9.80	8.56E-03
		peptide secretion	9	3.40	8.58E-03
		regulation of cellular response to oxidative stress	6	6.67	8.58E-03
		cellular response to UV	6	6.67	8.58E-03

	Category	Term	Count	Percent(%)	p-value
**Gene ontology**	**Biological process**	odontogenesis of dentin-containing tooth	6	6.67	8.58E-03
		negative regulation of cell-cell adhesion	8	4.04	8.58E-03
		tyrosine phosphorylation of STAT protein	6	6.59	9.15E-03
		nitrogen compound metabolic process	67	0.66	9.34E-03
		inositol lipid-mediated signaling	8	3.98	9.59E-03
		positive regulation of coagulation	4	16.67	9.66E-03
		positive regulation of cytokine production involved in inflammatory response	4	16.67	9.66E-03
		regulation of membrane protein ectodomain proteolysis	4	16.67	9.66E-03
		regulation of protein localization to nucleus	7	4.93	9.73E-03
		regulation of carbohydrate catabolic process	6	6.52	9.75E-03
		positive regulation of establishment of protein localization	10	2.90	9.87E-03
		regulation of mitotic cell cycle	13	2.12	1.03E-02
		gene expression	49	0.79	1.09E-02
		negative regulation of leukocyte proliferation	6	6.38	1.11E-02
		positive regulation of interleukin-6 production	6	6.38	1.11E-02
		regulation of hormone secretion	9	3.28	1.13E-02
		dsRNA processing	5	9.26	1.14E-02
		production of small RNA involved in gene silencing by RNA	5	9.26	1.14E-02
		positive regulation of amyloid precursor protein catabolic process	4	16.00	1.15E-02
		negative regulation of lipid catabolic process	4	16.00	1.15E-02
		positive regulation of glycolytic process	4	16.00	1.15E-02
		negative regulation of muscle contraction	4	16.00	1.15E-02
		macromolecule localization	32	1.02	1.15E-02
		positive regulation of peptidase activity	8	3.88	1.15E-02
		cellular nitrogen compound biosynthetic process	43	0.85	1.17E-02

	Category	Term	Count	Percent(%)	p-value
**Gene ontology**	**Biological process**	execution phase of apoptosis	6	6.32	1.18E-02
		regulation of endopeptidase activity	11	2.52	1.23E-02
		response to cAMP	6	6.25	1.25E-02
		negative regulation of blood coagulation	5	9.09	1.25E-02
		ovarian follicle development	5	9.09	1.25E-02
		embryo development	17	1.61	1.27E-02
		response to ionizing radiation	7	4.73	1.28E-02
		female gonad development	6	6.19	1.33E-02
		osteoclast differentiation	6	6.19	1.33E-02
		apoptotic DNA fragmentation	4	15.38	1.35E-02
		negative regulation of heart contraction	4	15.38	1.35E-02
		astrocyte activation	4	15.38	1.35E-02
		positive regulation of osteoclast differentiation	4	15.38	1.35E-02
		decidualization	4	15.38	1.35E-02
		regulation of lipid storage	5	8.93	1.37E-02
		negative regulation of hemostasis	5	8.93	1.37E-02
		cellular oxidant detoxification	6	6.06	1.49E-02
		positive regulation of neuron death	6	6.06	1.49E-02
		cell death in response to oxidative stress	6	6.06	1.49E-02
		regulation of response to oxidative stress	6	6.06	1.49E-02
		glial cell proliferation	5	8.77	1.49E-02
		positive regulation of cellular component organization	18	1.51	1.53E-02
		negative regulation of blood circulation	4	14.81	1.58E-02
		immunoglobulin production	6	6.00	1.58E-02
		regulation of chemokine production	6	6.00	1.58E-02

	Category	Term	Count	Percent(%)	p-value
**Gene ontology**	**Biological process**	negative regulation of intrinsic apoptotic signaling pathway	6	6.00	1.58E-02
		positive regulation of oxidoreductase activity	5	8.62	1.63E-02
		insulin secretion	8	3.70	1.63E-02
		adaptive immune response based on somatic recombination of immune receptors built from immunoglobulin superfamily domains	10	2.73	1.66E-02
		negative regulation of phosphate metabolic process	12	2.21	1.67E-02
		chemokine production	6	5.94	1.68E-02
		negative regulation of phosphorus metabolic process	12	2.21	1.70E-02
		release of cytochrome c from mitochondria	5	8.47	1.77E-02
		development of primary female sexual characteristics	6	5.88	1.78E-02
		neutrophil chemotaxis	6	5.88	1.78E-02
		endothelial cell migration	9	3.09	1.83E-02
		regulation of oxidative stress-induced intrinsic apoptotic signaling pathway	4	14.29	1.84E-02
		positive regulation of vascular endothelial growth factor production	4	14.29	1.84E-02
		positive regulation of heart rate	4	14.29	1.84E-02
		response to immobilization stress	4	14.29	1.84E-02
		regulation of lipid catabolic process	5	8.33	1.93E-02
		response to insulin	9	3.07	1.93E-02
		positive regulation of T cell proliferation	6	5.77	1.99E-02
		response to heat	7	4.40	2.05E-02
		maintenance of location in cell	8	3.59	2.06E-02
		membrane protein proteolysis	5	8.20	2.09E-02
		positive regulation of interleukin-1 beta production	5	8.20	2.09E-02
		negative regulation of coagulation	5	8.20	2.09E-02
		regulation of fatty acid metabolic process	6	5.71	2.10E-02

	Category	Term	Count	Percent(%)	p-value
Gene ontology	Biological process	interleukin-1-mediated signaling pathway	6	5.71	2.10E-02
		negative regulation of gene expression	20	1.36	2.11E-02
		T-helper 17 cell differentiation	4	13.79	2.12E-02
		regulation of macroautophagy	7	4.35	2.22E-02
		vascular endothelial growth factor production	5	8.06	2.27E-02
		G protein-coupled receptor signaling pathway, coupled to cyclic nucleotide second messenger	5	8.06	2.27E-02
		negative regulation of lipid metabolic process	6	5.61	2.34E-02
		regulation of cellular protein localization	12	2.13	2.40E-02
		regulation of blood vessel endothelial cell migration	7	4.29	2.41E-02
		positive regulation of immune effector process	8	3.51	2.42E-02
		response to angiotensin	4	13.33	2.44E-02
		lipid biosynthetic process	14	1.82	2.45E-02
		regulation of receptor signaling pathway via JAK-STAT	6	5.56	2.47E-02
		humoral immune response	10	2.61	2.47E-02
		protein transport	22	1.25	2.48E-02
		biomineral tissue development	7	4.27	2.51E-02
		regulation of production of molecular mediator of immune response	7	4.27	2.51E-02
		vitamin D biosynthetic process	3	30.00	2.56E-02
		negative regulation of fibrinolysis	3	30.00	2.56E-02
		negative regulation by symbiont of host apoptotic process	3	30.00	2.56E-02
		regulation of calcium ion transmembrane transport	7	4.24	2.61E-02
		developmental process involved in reproduction	16	1.60	2.65E-02
		regulation of multicellular organism growth	5	7.81	2.65E-02
		biomineralization	7	4.22	2.71E-02
		fat cell differentiation	8	3.45	2.74E-02

	Category	Term	Count	Percent(%)	p-value
**Gene ontology**	**Biological process**	phagocytosis	10	2.58	2.76E-02
		organic hydroxy compound metabolic process	12	2.10	2.77E-02
		negative regulation of ion transport	7	4.19	2.82E-02
		calcium ion transport into cytosol	7	4.19	2.82E-02
		leukocyte mediated immunity	15	1.69	2.83E-02
		regulation of smooth muscle contraction	5	7.69	2.86E-02
		regulation of osteoclast differentiation	5	7.69	2.86E-02
		regulation of morphogenesis of an epithelium	5	7.69	2.86E-02
		negative regulation of cell adhesion	9	2.92	2.88E-02
		regulation of nucleocytoplasmic transport	6	5.41	2.89E-02
		positive regulation of mononuclear cell migration	5	7.58	3.09E-02
		regulation of B cell proliferation	5	7.58	3.09E-02
		long-chain fatty acid biosynthetic process	4	12.50	3.18E-02
		positive regulation of endothelial cell proliferation	6	5.31	3.20E-02
		Fc receptor signaling pathway	8	3.36	3.30E-02
		protein localization	28	1.05	3.35E-02
		long-chain fatty acid metabolic process	6	5.26	3.36E-02
		regulation of interleukin-6 production	7	4.07	3.42E-02
		interleukin-6 production	7	4.07	3.42E-02
		positive regulation of MHC class II biosynthetic process	3	27.27	3.51E-02
		cellular response to UV-A	3	27.27	3.51E-02
		modulation by symbiont of host apoptotic process	3	27.27	3.51E-02
		negative regulation by symbiont of host programmed cell death	3	27.27	3.51E-02
		regulation of MAP kinase activity	9	2.85	3.53E-02
		response to amino acid	6	5.22	3.54E-02

	Category	Term	Count	Percent(%)	p-value
Gene ontology	Biological process	negative regulation of cellular protein metabolic process	17	1.49	3.56E-02
		CD4-positive, alpha-beta T cell differentiation involved in immune response	5	7.35	3.57E-02
		DNA catabolic process, endonucleolytic	4	12.12	3.60E-02
		T-helper 17 type immune response	4	12.12	3.60E-02
		hormone secretion	9	2.84	3.62E-02
		positive regulation of cell cycle	9	2.84	3.62E-02
		regulation of protein catabolic process	10	2.49	3.67E-02
		xenobiotic metabolic process	6	5.17	3.71E-02
		glycolytic process	6	5.17	3.71E-02
		female sex differentiation	6	5.17	3.71E-02
		biological process involved in interaction with host	8	3.29	3.84E-02
		alpha-beta T cell differentiation involved in immune response	5	7.25	3.84E-02
		alpha-beta T cell activation involved in immune response	5	7.25	3.84E-02
		ATP generation from ADP	6	5.13	3.90E-02
		regulation of gene silencing by miRNA	6	5.13	3.90E-02
		regulation of endothelial cell migration	8	3.28	3.95E-02
		regulation of superoxide metabolic process	4	11.76	4.07E-02
		regulation of receptor signaling pathway via STAT	6	5.08	4.10E-02
		cellular response to drug	5	7.14	4.12E-02
		regulation of blood coagulation	5	7.14	4.12E-02
		epithelial cell differentiation	14	1.73	4.27E-02
		regulation of posttranscriptional gene silencing	6	5.04	4.30E-02
		unsaturated fatty acid metabolic process	6	5.04	4.30E-02
		heart development	12	2.01	4.38E-02
		positive regulation of chemokine production	5	7.04	4.41E-02

	Category	Term	Count	Percent(%)	p-value
Gene ontology	Biological process	regulation of gene silencing by RNA	6	5.00	4.51E-02
		inorganic cation transmembrane transport	14	1.72	4.58E-02
		negative regulation of extrinsic apoptotic signaling pathway in absence of ligand	4	11.43	4.58E-02
		negative regulation of signal transduction in absence of ligand	4	11.43	4.58E-02
		apoptotic nuclear changes	4	11.43	4.58E-02
		striated muscle contraction	7	3.89	4.59E-02
		embryonic morphogenesis	12	2.00	4.60E-02
		calcium ion transmembrane transport	9	2.75	4.63E-02
		hormone transport	9	2.75	4.63E-02
		regulation of apoptotic DNA fragmentation	3	25.00	4.66E-02
		xenobiotic catabolic process	3	25.00	4.66E-02
		cellular response to xenobiotic stimulus	6	4.96	4.72E-02
		gland morphogenesis	6	4.96	4.72E-02
		regulation of synaptic transmission, glutamatergic	5	6.94	4.73E-02
		positive regulation of interleukin-1 production	5	6.94	4.73E-02
		regulation of hemostasis	5	6.94	4.73E-02
		positive regulation of the force of heart contraction by epinephrine-norepinephrine	2	100.00	4.82E-02
		negative regulation of synaptic transmission, dopaminergic	2	100.00	4.82E-02
		positive regulation of vitamin D biosynthetic process	2	100.00	4.82E-02
		neutrophil migration	6	4.92	4.95E-02
	Cellular components	membrane microdomain	19	5.60	1.20E-13
		membrane raft	19	5.60	1.20E-13
		plasma membrane region	25	2.02	1.95E-08
		intrinsic component of plasma membrane	28	1.62	1.56E-07
		cell periphery	55	0.89	1.75E-07

	Category	Term	Count	Percent(%)	p-value
**Gene ontology**	**Cellular components**	integral component of plasma membrane	27	1.64	2.70E-07
		caveola	8	9.64	8.82E-07
		plasma membrane	51	0.90	1.35E-06
		integral component of presynaptic membrane	7	10.00	6.89E-06
		plasma membrane raft	8	6.96	1.18E-05
		intrinsic component of presynaptic membrane	7	8.86	1.61E-05
		extracellular space	37	1.03	3.25E-05
		presynaptic membrane	8	5.41	8.35E-05
		integral component of postsynaptic membrane	7	5.93	2.56E-04
		intrinsic component of postsynaptic membrane	7	5.65	3.57E-04
		cell junction	25	1.19	7.43E-04
		integral component of synaptic membrane	7	4.61	1.39E-03
		extracellular region	39	0.85	1.72E-03
		axon	13	1.97	1.78E-03
		cell surface	15	1.67	2.25E-03
		intrinsic component of synaptic membrane	7	4.22	2.48E-03
		synapse	18	1.34	5.58E-03
		apical part of cell	10	2.31	5.90E-03
		endomembrane system	38	0.82	7.48E-03
		neuron projection	18	1.30	7.94E-03
		apical plasma membrane	9	2.47	9.22E-03
		nuclear envelope	10	2.12	1.20E-02
		neuron projection cytoplasm	5	5.38	1.61E-02
		plasma membrane bounded cell projection	23	1.03	2.08E-02
		presynapse	10	1.94	2.53E-02

	Category	Term	Count	Percent(%)	p-value
**Gene ontology**	**Cellular components**	organelle envelope	16	1.29	2.72E-02
		envelope	16	1.29	2.72E-02
		spine apparatus	2	50.00	3.21E-02
		transcription factor AP-1 complex	2	50.00	3.21E-02
		cell projection	23	0.98	4.15E-02
	**Molecular function**	identical protein binding	36	1.75	4.82E-11
		cytokine receptor binding	15	5.47	6.74E-10
		enzyme binding	33	1.61	7.60E-09
		protein dimerization activity	24	2.24	1.59E-08
		adrenergic receptor activity	5	50.00	2.12E-07
		receptor ligand activity	16	3.23	2.98E-07
		cytokine activity	12	5.11	3.18E-07
		signaling receptor activator activity	16	3.19	3.65E-07
		protein homodimerization activity	18	2.65	5.21E-07
		G protein-coupled amine receptor activity	7	15.22	7.05E-07
		signaling receptor binding	26	1.68	1.05E-06
		alpha1-adrenergic receptor activity	3	100.00	4.62E-05
		nuclear receptor activity	6	11.11	7.89E-05
		ligand-activated transcription factor activity	6	11.11	7.89E-05
		transcription factor binding	14	2.52	8.96E-05
		RNA polymerase II-specific DNA-binding transcription factor binding	9	4.59	1.26E-04
		heme binding	8	5.56	1.44E-04
		tetrapyrrole binding	8	5.19	2.40E-04
		protein binding	82	0.56	3.62E-04
		molecular transducer activity	22	1.42	4.56E-04

	Category	Term	Count	Percent(%)	p-value
Gene ontology	Molecular function	signaling receptor activity	22	1.42	4.56E-04
		DNA-binding transcription factor binding	9	3.85	5.58E-04
		alpha-adrenergic receptor activity	3	50.00	9.15E-04
		protein domain specific binding	14	1.98	1.54E-03
		phosphatase binding	8	4.02	1.65E-03
		nitric-oxide synthase regulator activity	3	33.33	3.80E-03
		scaffold protein binding	5	8.20	4.19E-03
		growth factor activity	7	4.27	4.78E-03
		protein kinase binding	13	1.92	5.11E-03
		protein-containing complex binding	18	1.38	8.29E-03
		chemokine receptor binding	5	6.94	9.48E-03
		serotonin binding	3	25.00	9.87E-03
		amine binding	3	25.00	9.87E-03
		prostaglandin-endoperoxide synthase activity	2	100.00	1.05E-02
		acetylcholinesterase activity	2	100.00	1.05E-02
		MAP kinase activity	3	21.43	1.62E-02
		efflux transmembrane transporter activity	3	21.43	1.62E-02
		DNA-binding transcription activator activity, RNA polymerase II-specific	10	2.24	1.63E-02
		kinase binding	13	1.72	1.66E-02
		protein heterodimerization activity	9	2.51	1.76E-02
		DNA-binding transcription activator activity	10	2.21	1.83E-02
		antioxidant activity	5	5.75	2.37E-02
		kinase regulator activity	7	3.20	3.07E-02
		nitric-oxide synthase activity	2	66.67	3.15E-02
		IkappaB kinase activity	2	66.67	3.15E-02

	Category	Term	Count	Percent(%)	p-value
**Gene ontology**	**Molecular function**	cholinesterase activity	2	66.67	3.15E-02
		vitamin D 24-hydroxylase activity	2	66.67	3.15E-02
		CXCR chemokine receptor binding	3	16.67	3.59E-02
		chemokine activity	4	8.16	3.64E-02
		oxidoreductase activity, acting on paired donors, with incorporation or reduction of molecular oxygen, NAD(P)H as one donor, and incorporation of one atom of oxygen	4	8.16	3.64E-02
		peptide binding	8	2.56	4.23E-02
		peroxidase activity	4	7.55	4.96E-02
**Pathway**	**KEGG**	AGE-RAGE signaling pathway in diabetic complications	21	21.00	3.15E-21
		IL-17 signaling pathway	20	21.74	1.85E-20
		Chagas disease	20	19.80	1.39E-19
		Pathways in cancer	34	6.43	2.24E-18
		Lipid and atherosclerosis	24	11.21	9.42E-18
		TNF signaling pathway	18	16.07	9.49E-16
		Toxoplasmosis	17	15.60	1.44E-14
		Hepatitis B	19	11.73	4.69E-14
		Th17 cell differentiation	16	15.38	1.54E-13
		Leishmaniasis	14	19.44	3.38E-13
		Human cytomegalovirus infection	20	8.97	1.39E-12
		Toll-like receptor signaling pathway	15	14.71	2.56E-12
		Coronavirus disease - COVID-19	20	8.66	2.74E-12
		T cell receptor signaling pathway	15	14.56	2.97E-12
		Yersinia infection	16	11.76	1.19E-11
		Fluid shear stress and atherosclerosis	16	11.59	1.51E-11
		Kaposi sarcoma-associated herpesvirus infection	18	9.33	1.77E-11

	Category	Term	Count	Percent(%)	p-value
Pathway	KEGG	Pertussis	13	17.11	2.04E-11
		Pancreatic cancer	13	17.11	2.04E-11
		C-type lectin receptor signaling pathway	14	13.46	7.07E-11
		Hepatitis C	16	10.19	1.15E-10
		Influenza A	16	9.47	3.64E-10
		Osteoclast differentiation	14	11.20	9.29E-10
		Inflammatory bowel disease	11	17.74	1.06E-09
		Human T-cell leukemia virus 1 infection	17	7.87	1.43E-09
		HIF-1 signaling pathway	13	11.93	2.42E-09
		PD-L1 expression and PD-1 checkpoint pathway in cancer	12	13.48	3.42E-09
		Pathogenic Escherichia coli infection	16	8.16	3.53E-09
		Measles	14	10.07	3.99E-09
		Endocrine resistance	12	12.63	7.53E-09
		Chronic myeloid leukemia	11	14.47	1.08E-08
		Non-alcoholic fatty liver disease	14	9.33	1.12E-08
		Salmonella infection	17	6.83	1.37E-08
		Amoebiasis	12	11.88	1.57E-08
		Relaxin signaling pathway	13	10.08	2.09E-08
		MAPK signaling pathway	18	6.12	2.20E-08
		Antifolate resistance	8	25.81	3.18E-08
		Apoptosis	13	9.56	4.08E-08
		Colorectal cancer	11	12.79	4.25E-08
		Th1 and Th2 cell differentiation	11	12.36	6.20E-08
		Malaria	9	18.37	6.35E-08
		Proteoglycans in cancer	15	7.32	7.27E-08

	Category	Term	Count	Percent(%)	p-value
Pathway	KEGG	Tuberculosis	14	8.00	8.81E-08
		Bladder cancer	8	19.51	3.56E-07
		Rheumatoid arthritis	10	11.36	8.98E-07
		Human immunodeficiency virus 1 infection	14	6.67	9.49E-07
		NOD-like receptor signaling pathway	13	7.34	1.05E-06
		Acute myeloid leukemia	9	13.43	1.15E-06
		Small cell lung cancer	10	10.87	1.39E-06
		Sphingolipid signaling pathway	11	9.24	1.42E-06
		Chemokine signaling pathway	13	6.84	2.47E-06
		NF-kappa B signaling pathway	10	9.80	3.80E-06
		FoxO signaling pathway	11	8.40	3.89E-06
		Epstein-Barr virus infection	13	6.57	4.04E-06
		Hepatocellular carcinoma	12	7.23	4.84E-06
		B cell receptor signaling pathway	9	11.39	5.03E-06
		EGFR tyrosine kinase inhibitor resistance	9	11.39	5.03E-06
		Estrogen signaling pathway	11	8.03	6.20E-06
		Insulin resistance	10	9.26	6.59E-06
		Shigellosis	14	5.69	7.06E-06
		Serotonergic synapse	10	8.93	9.33E-06
		Adipocytokine signaling pathway	8	11.59	2.50E-05
		Prolactin signaling pathway	8	11.43	2.81E-05
		Epithelial cell signaling in Helicobacter pylori infection	8	11.43	2.81E-05
		Prostate cancer	9	9.28	3.04E-05
		Choline metabolism in cancer	9	9.18	3.32E-05
		Non-small cell lung cancer	8	11.11	3.50E-05

	Category	Term	Count	Percent(%)	p-value
**Pathway**	**KEGG**	Platinum drug resistance	8	11.11	3.50E-05
		cGMP-PKG signaling pathway	11	6.63	4.40E-05
		Allograft rejection	6	17.65	6.53E-05
		African trypanosomiasis	6	16.67	9.31E-05
		PI3K-Akt signaling pathway	15	4.25	1.02E-04
		ErbB signaling pathway	8	9.52	1.16E-04
		VEGF signaling pathway	7	11.86	1.24E-04
		Adrenergic signaling in cardiomyocytes	10	6.67	1.43E-04
		Cellular senescence	10	6.41	2.05E-04
		Calcium signaling pathway	12	5.02	2.48E-04
		JAK-STAT signaling pathway	10	6.17	2.88E-04
		Cytokine-cytokine receptor interaction	13	4.44	3.62E-04
		Intestinal immune network for IgA production	6	13.33	3.63E-04
		Viral protein interaction with cytokine and cytokine receptor	8	8.16	3.76E-04
		RIG-I-like receptor signaling pathway	7	10.00	3.98E-04
		cAMP signaling pathway	11	5.09	5.84E-04
		Glioma	7	9.33	6.34E-04
		Alzheimer disease	14	3.79	9.10E-04
		Breast cancer	9	6.12	9.89E-04
		Cholinergic synapse	8	7.08	1.09E-03
		Ras signaling pathway	11	4.76	1.11E-03
		Transcriptional misregulation in cancer	10	5.24	1.25E-03
		Regulation of lipolysis in adipocytes	6	10.71	1.33E-03
		Oxytocin signaling pathway	9	5.84	1.44E-03
		Legionellosis	6	10.53	1.47E-03

	Category	Term	Count	Percent(%)	p-value
Pathway	KEGG	Neurotrophin signaling pathway	8	6.72	1.59E-03
		Endometrial cancer	6	10.34	1.63E-03
		Gap junction	7	7.95	1.83E-03
		Thyroid cancer	5	13.51	2.26E-03
		Graft-versus-host disease	5	13.51	2.26E-03
		Salivary secretion	7	7.69	2.28E-03
		Cytosolic DNA-sensing pathway	6	9.68	2.40E-03
		GnRH signaling pathway	7	7.53	2.63E-03
		MicroRNAs in cancer	12	3.87	3.45E-03
		Pathways of neurodegeneration - multiple diseases	15	3.16	3.73E-03
		Fc epsilon RI signaling pathway	6	8.96	3.75E-03
		Renal cell carcinoma	6	8.82	4.09E-03
		Central carbon metabolism in cancer	6	8.57	4.82E-03
		Melanoma	6	8.33	5.66E-03
		Type II diabetes mellitus	5	10.87	6.62E-03
		Asthma	4	14.81	1.06E-02
		Diabetic cardiomyopathy	9	4.43	1.25E-02
		Growth hormone synthesis, secretion and action	7	5.88	1.27E-02
		Thyroid hormone signaling pathway	7	5.79	1.41E-02
		Platelet activation	7	5.65	1.64E-02
		Apoptosis - multiple species	4	12.50	2.09E-02
		TGF-beta signaling pathway	6	6.45	2.35E-02
		Human papillomavirus infection	11	3.32	2.85E-02
		Apelin signaling pathway	7	5.15	2.90E-02
		Neuroactive ligand-receptor interaction	11	3.24	3.58E-02

	Category	Term	Count	Percent(%)	p-value
**Pathway**	**KEGG**	Mitophagy - animal	5	7.35	4.26E-02
		Phospholipase D signaling pathway	7	4.76	4.65E-02
		Gastric cancer	7	4.73	4.84E-02
		Type I diabetes mellitus	4	10.00	5.00E-02
